# Iron‐Based Catalysts on Tailored Alumina Supports for CO_*x*_‐Free Methane Decomposition to H_2_ and Carbon

**DOI:** 10.1002/open.70282

**Published:** 2026-09-01

**Authors:** Hajer S. Alturki, Seham S. Alterary, Abdulaziz A. M. Abahussain, Ahmed I. Osman, Mohammed O. Bayazed, Ahmed A. Ibrahim, Naif Alarifi, Fahad S. Almubaddel, Alaaddin M. M. Saeed, Rawesh Kumar, Ahmed S. Al‐Fatesh

**Affiliations:** ^1^ Department of Chemistry College of Science King Saud University Riyadh Saudi Arabia; ^2^ Department of Chemical Engineering College of Engineering King Saud University (KSU) Riyadh Saudi Arabia; ^3^ School of Sciences, Psychology, Arts and Humanities, Computing Engineering & Sports Canterbury Christ Church University Canterbury UK; ^4^ Institute of Refining and Petrochemicals Technologies, King Abdul Aziz City for Science and Technology (KACST) Riyadh Saudi Arabia; ^5^ UNILAB State Key Laboratory of Chemical Engineering East China University of Science and Technology Shanghai China; ^6^ Department of Chemistry Patna University Bihar India

**Keywords:** Fe‐based catalyst, hydrogen yield, metal oxide‐Modified alumina, methane decomposition, multi‐walled carbon nanotubes

## Abstract

Methane decomposition is a CO_*x*_‐free process for hydrogen generation that also produces valuable carbon nanostructures. The work herein focused on Fe‐based catalysts supported on some common supports (Al_2_O_3_, ZrO_2_, SiO_2_) and on alumina modified by metal oxides (10SiAl, 10ZrAl, 10YAl, 10CeAl). The prepared catalysts were characterized in detail by XRD, BET, TEM, TPR, TGA, and Raman spectroscopy. Among the single‐component supports, the 20Fe/Al_2_O_3_ (Fe‐Al) catalyst exhibited the highest initial activity, with 69.4% CH_4_ conversion. Among all the catalysts studied, Fe‐10ZrAl showed the best catalytic activity with a maximum CH_4_ conversion (76.5%) and H_2_ yield (74.1%). Furthermore, during regeneration cycles, the catalyst maintained CH_4_ conversion (90%) without detrimental carbon encapsulation. The performance of Fe‐10ZrAl is attributed to the synergistic effect of the ZrO_2_ modifier which improves strong metal–support interactions, stabilizes active iron nanoparticles from thermal sintering, and allows a highly reversible in‐situ carbide cycle. The Fe‐10ZrAl catalyst is the most active, followed by the Fe‐10YAl catalyst, owing to its improved reducibility. Our work demonstrates the key role of modified alumina structures for sustainable co‐production of hydrogen and nanomaterials.

## Graphical abstract

Fe‐10ZrAl catalyst enables catalytic methane decomposition into CO*_x_*‐free H_2_ and solid carbon. ZrO_2_‐modified alumina improves Fe particle stabilization, reducibility, and carbon growth behavior, giving the best performance among the tested catalysts with 76.5% CH_4_ conversion and 74.1% H_2_ yield at 800 °C.

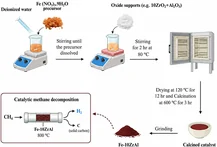

## 1 Introduction

As a potential solution to environmental and energy issues, hydrogen energy stands out as a leading competitor. It offers a sustainable and eco‐friendly alternative to conventional energy sources [1]. Since hydrogen has a higher gravimetric energy content than fossil fuels, it is a suitable candidate for reducing reliance on them [2, 3]. It is a clean energy carrier with various industrial applications, such as ammonia production, hydrogenation, and hydrodesulfurization [4]. Moreover, hydrogen can be used for electric power generation systems, encompassing fuel cells. These fuel cells employ electrochemical methods to convert the energy in hydrogen into electricity, with low emissions and a by‐product (heat) [5]. The most cost‐effective and widely used conventional technology for producing approximately 48% of the world's hydrogen supply is steam reforming of methane [6]. However, these processes generate CO_*x*_, along with hydrogen, which are detrimental to the environment [6]. Recently, catalytic decomposition of methane (CDM) technology has attracted more attention, as it can produce hydrogen with zero CO_2_ emissions [7]. Although CH_4_ is a greenhouse gas that contributes to global warming, it has a high H/C ratio, making it a necessary resource for hydrogen synthesis. Methane's popularity as a resource is further increased by its extensive availability in sources such as shale gas, natural gas, and industrial waste gases [8]. Four identical strong sp^3^‐hybridized C·H bonds with a bond energy of 434 kJ/mol make up the typical tetrahedral structure of CH_4_. Due to its stable structure, methane must be broken down at temperatures above 1200 °C. This endothermic reaction yields solid carbon and gaseous hydrogen in a single step, as indicated by Equation (I) [9].

(I) $${\mathrm{CH}}_{4(g)}\;\to \;C_{\left(s\right)}+2H_{2(g)}\;\text{Δ}H_{293}=74.8\;\text{KJ}/\mathrm{mol}$$

The use of suitable heterogeneous catalysts has proven to be a successful contribution in reducing the activation energy and decreasing the reaction time at lower temperatures (450–750 °C) [9, 10]. Metal‐based catalysts can form carbon nanotubes (CNTs), carbon nanofibers (CNFs), and graphitic nanolayers as by‐products under particular conditions, creating a commercially valuable material used in various fields [10, 11], including as additives in polymers and plastics for the automotive and construction sectors, as well as catalyst supports. Furthermore, it is anticipated that their use in electronics and energy storage will expand significantly in the future [12]. The low cost, high catalytic activity, and durability of catalysts based on transition metals (Ni, Co, Fe) have received considerable interest [13, 14]. Their partially filled 3d orbitals, which can accept electrons from methane's C·H bonds and facilitate its breakdown, are responsible for their catalytic activity [15]. Fe‐based catalysts are the best option among these metals, as they are more cost‐effective and environmentally friendly than Co‐ and Ni‐based catalysts [16, 17]. Even though Ni is more active than Fe in methane breakdown, Fe catalysts maintain stability and catalytic activity up to 700–1000 °C [16]. Iron catalysts have a longer catalyst lifetime because they maintain a better balance between carbon precipitation and diffusion rates [16]. It has previously been observed that 20 wt.% loading of Fe results in superior dispersion and higher catalytic activity [18].

Support material for catalysts significantly influences catalytic activity. High surface area, stability at high temperatures, and resilience to mechanical stress are all desirable qualities of an excellent support [10]. Moreover, the nature of the support affects the strength of the metal–support interaction and thus the distribution of the active component [19]. In the CDM process, metal oxide compounds such as SiO_2_ [20], MgO [21], ZrO_2_ [22], CeO_2_ [23], and Al_2_O_3_ [24] were employed to support Fe‐based catalysts. Due to the strong metal–support interaction, the increased dissociation of CH_4_ over stable active sites (metallic Fe) has made Fe/Al_2_O_3_ the subject of numerous prior studies. Fe_3_C is also an active site, which is created during the CDM reaction over the Fe/Al_2_O_3_ catalyst [25]. Composite oxides are an effective approach to overcoming the drawbacks of supporting materials and improving catalyst efficiency [26]. Incorporating metal textural promoters such as TiO_2_ and MgO into Al_2_O_3_ was shown to boost the reaction's effectiveness [27, 28].

In the literature, metal‐incorporated ceria was found to selective in photoreduction [29, 30]. Research findings suggest that the addition of CeO_2_ to Al_2_O_3_ has been found to improve the dispersion of the active phase and induce the generation of oxygen vacancies, which enhanced the catalytic activity [31]. The incorporation of zirconia effectively inhibits alumina sintering, thereby enhancing the material's thermal stability at elevated temperatures [32]. Further studies reported that ZrO_2_ provides oxygen vacancies [33] and improves catalyst stability by suppressing carbon deposition and metal sintering under reaction conditions [34]. Although these studies were conducted on non‐iron‐based systems, the results regarding improved dispersion, stability, and carbonation resistance provide a valuable research perspective that could guide the future development of supports suitable for Fe‐based catalysts. From the reviewed literature, it is evident that Fe‐based catalysts offer a cost‐effective alternative to Ni and Co systems for methane decomposition but often suffer from rapid deactivation due to carbon encapsulation and sintering.

This study fills a gap in existing research by modifying the catalyst support and focusing on the effects of ZrO_2_‐ and Y_2_O_3_‐modifying Al_2_O_3_‐supported Fe catalysts. The primary focus of the present work is the production of high‐purity, CO_*x*_‐free hydrogen. This study reports the synthesis and evaluation of a series of 20% Fe‐based catalysts prepared by wet impregnation. Diverse supports of single oxides (Al_2_O_3_, ZrO_2_, and SiO_2_) and modified supports of binary oxides were synthesized from Al_2_O_3_ modified by 10% of (SiO_2_, CeO_2_, Y_2_O_3_, and ZrO_2_). The modification is intended to achieve an optimal, highly dispersed distribution of both metallic Fe and Fe_3_C active phases, thereby enhancing overall methane decomposition efficiency. The synthesized catalysts and spent catalysts were thoroughly characterized using a combination of state‐of‐the‐art bulk‐sensitive and surface‐sensitive analytical techniques to strengthen the findings.

## 2 Experimental

### 2.1 Materials

Alumina [SA‐6175; acquired from Norton] and iron nitrate [Fe (NO_3_)_3_·9H_2_O; 98% pure; analytical grades obtained from Loba Chemie] were used without additional purification. Silica [SS‐5231; 75‐100% bought from Norton], zirconia [Daiichi Kigenso Kagaku Kogyo Co., Ltd., Osaka, Japan], yttria [Y_2_O_3_; 99.95% pure obtained from MKnano], and ceria [CeO_2_; 99.9% pure purchased from MKnano]. The Millipore water purification system (Merck KGaA, Darmstadt, Germany) was utilized to acquire distilled water. All synthesized samples were designed for an Fe loading of a nominal 20 wt.% based on stoichiometric precursor calculations.

### 2.2 Catalyst Preparation

**FIGURE 1:**
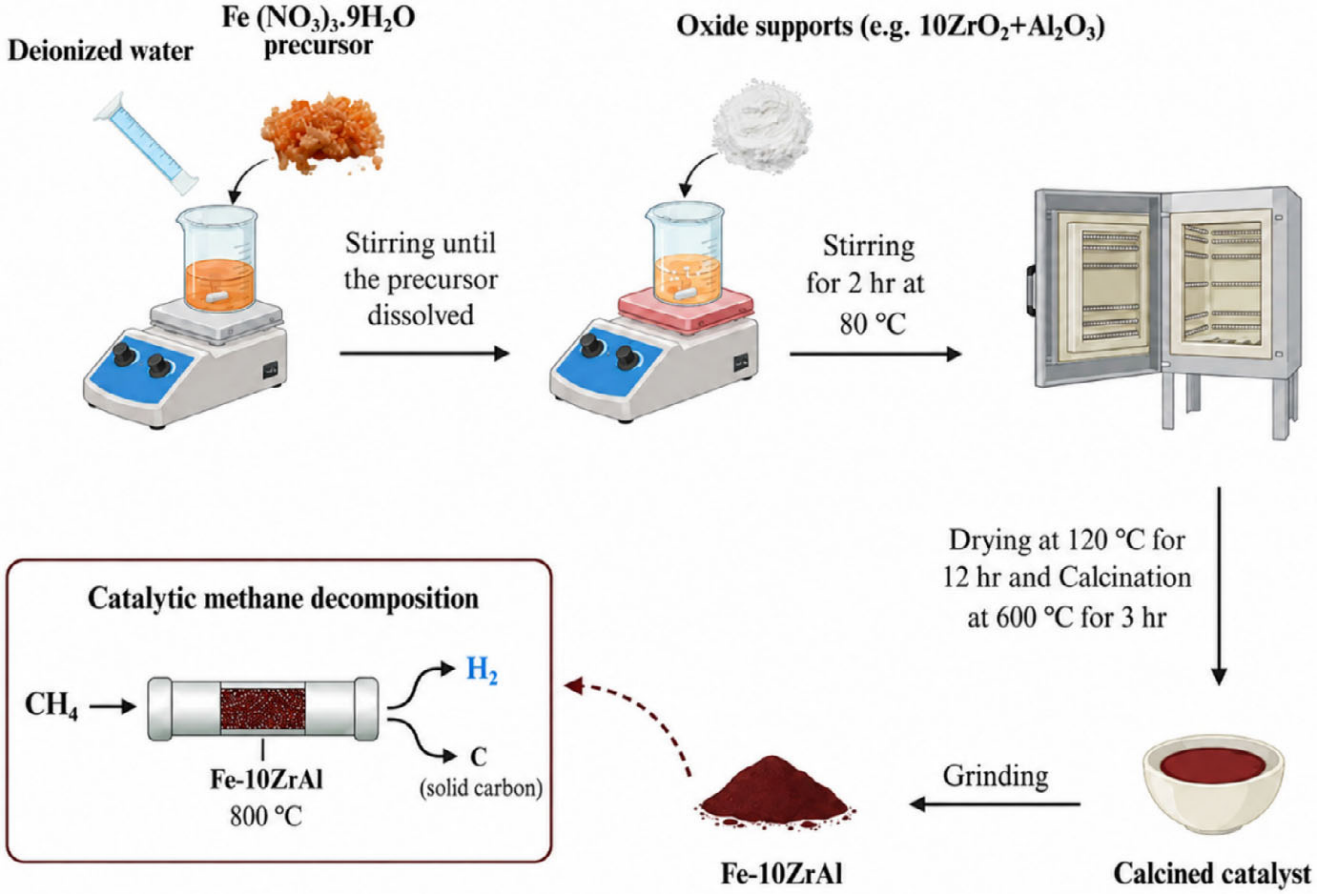
The scheme of the catalyst preparation.

The catalysts were prepared by the wet impregnation method with 20 mL of distilled water. The target iron loading was set at 20 wt.%, and the rest was made up of support (80 wt.%). In the first stage of our study, we prepared and characterized three single‐oxide supports (SiO_2_, ZrO_2_, and Al_2_O_3_) for the stabilization of 20 wt.% Fe. To this end, the Fe‐nitrate solution was incorporated into suitable supports and mixed at 80 °C for 2 h to ensure complete mixing and homogeneous distribution of the precursor in the support material. The catalysts were dried at 120 °C for 12 h to remove all the moisture. Subsequently, the catalysts were calcined at 600 °C in air for 3 h. Al_2_O_3_ was found to be the best support for Fe. In the second step, the Al_2_O_3_ support was modified by the addition of 10 wt.% of SiO_2_, ZrO_2_, Y_2_O_3_, and CeO_2_. The second stage was carried out on commercial composite supports consisting of 90 wt.% Al_2_O_3_ and 10 wt.% of the corresponding modifying oxides SiO_2_, ZrO_2_, Y_2_O_3_, and CeO_2_. The modified Fe‐based catalysts (Fe‐10SiAl, Fe‐10ZrAl, Fe‐10CeAl, and Fe‐10YAl) were synthesized by direct wet impregnation of the commercial composite supports with the iron precursor. The resulting catalyst materials were ground into a fine powder for subsequent catalytic activity evaluation. Figure 1 describes the catalyst preparation scheme for Fe‐10ZrAl, and a similar scheme was used to synthesize all the modified catalysts. For pure alumina, the catalysts are referred to as Fe‐Al. Alumina supports containing 10% ceria, silica, zirconia, or yttria are called Fe‐10CeAl, Fe‐10SiAl, Fe‐10ZrAl, and Fe‐10YAl, respectively. The catalyst and carbon characterization are inserted in the Support Information as S1.

### 2.3 Catalytic Activity Test

**FIGURE 2:**
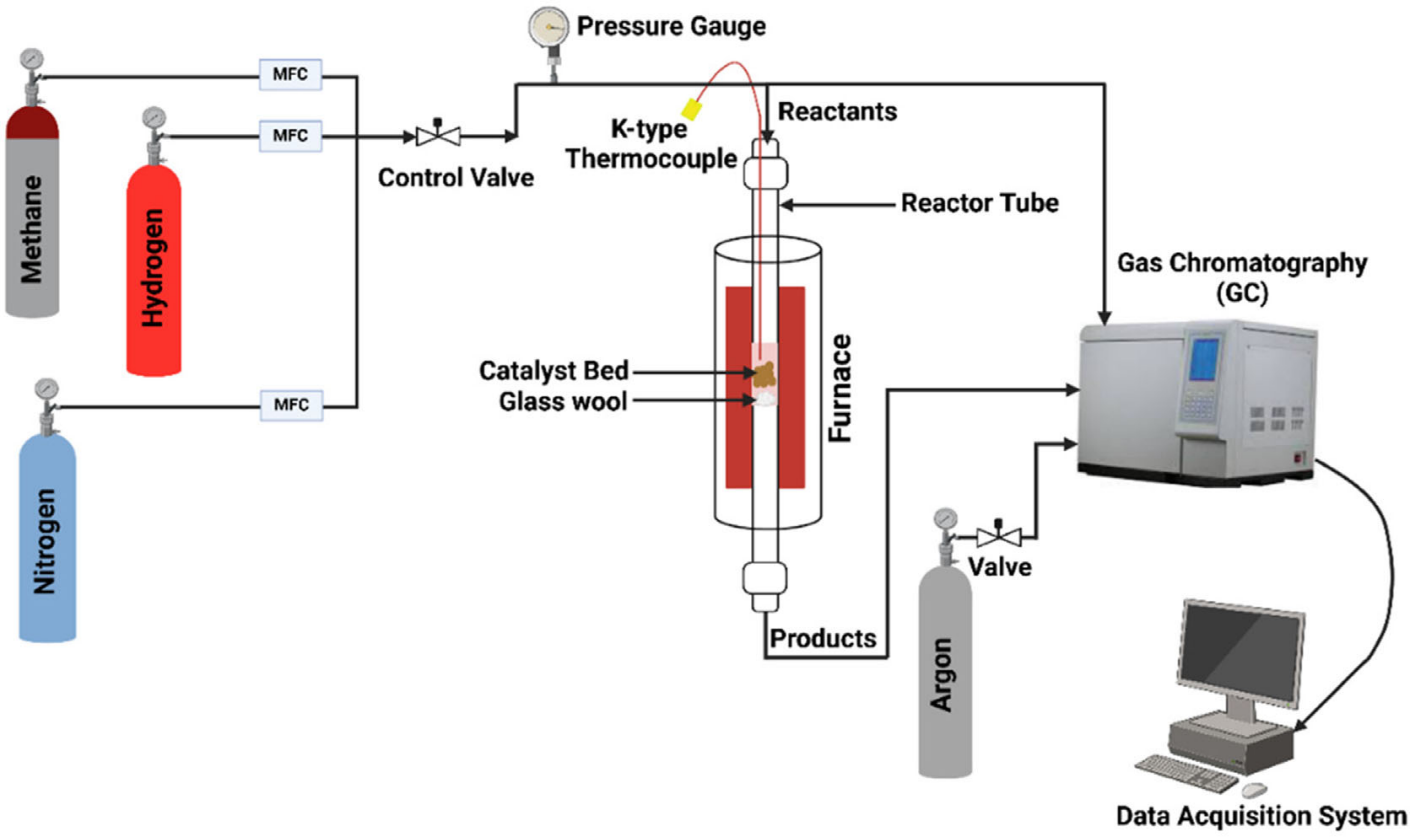
Schematic diagram of the experimental setup for catalytic hydrogen production. Reaction conditions: Tr = 800 °C, *p* = 1 atm, CH_4_:N_2_ feed ratio = 2:1, and GHSV = 3600 mLg^−1^h^−1^.

Fe‐based catalysts were evaluated for the CDM reaction at atmospheric pressure in a vertical, fixed‐bed, stainless‐steel tubular microreactor (PID Eng & Tech micro‐activity reference) with a length of 13 cm and an inner diameter of 9.1 mm. The reactor was placed in a tube furnace. The reactor temperature was measured using a K‐type thermocouple located axially in the catalyst bed. Figure 2 presents a detailed schematic diagram of this reactor setup. First, the reactor was loaded with 0.3 g of calcined catalyst on a glass wool substrate. The new catalyst was reduced in a flow of hydrogen (40 mL/min) for 60 min at 700 °C. Then a flow of N_2_ of 15 mL/min was used to purge any remaining H_2_. At the same time, the reactor temperature was raised to the required reaction temperature of 800 °C. The feed gas mixture was fed at a total flow rate of 18 mL/min and an equivalent space velocity of 3600 mL h^−1^ gcat^−1^ with a ratio of CH_4_ to N_2_ of 2. The composition of the reactor effluent was studied using a gas chromatography apparatus (GC‐Shimadzu 2014) with a thermal conductivity detector (TCD). Gas chromatography was performed on a packed column. The conversion yields of CH_4_ with hydrogen and carbon were calculated using the formulas given below (Equations (1)–(3)).

(1) $$X_{{\mathrm{CH}}_{4}}\left(\%\right)=\frac{\left[{\mathrm{CH}}_{4,\mathrm{in}}-{\mathrm{CH}}_{4,\mathrm{out}}\right]}{\left[{\mathrm{CH}}_{4,\mathrm{in}}\right]}\times 100$$

(2) $$Y_{H_{2}}\left(\%\right)=\frac{\left[H_{2,\mathrm{out}}\right]}{\left[2\times {\mathrm{CH}}_{4,\mathrm{in}}\right]}\times 100$$

(3) $$\text{Carbon yield}\left(\%\right)=\frac{\left[W_{p}-W_{\mathrm{cat}}\right]}{\left[W_{\mathrm{cat}}\right]}\times 100$$

where [CH_4,out_] and [H_2,out_] represent the methane and hydrogen output, respectively, and [CH_4,in_] symbolizes the feed's original methane. *W*_P_ is the weight of the product after the reaction, and *W*_cat_ is the weight of the fresh catalyst.

## 3 Results and Analysis

### 3.1 Catalytic Activity Evaluation

**FIGURE 3:**
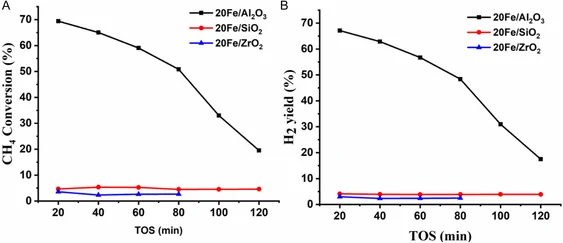
Catalytic activity results of Iron catalyst over different support: (A) CH_4_ conversion (%) vs TOS; (B) H_2_ yield (%) vs TOS (at 800 °C reaction temperature, 1 atm pressure, CH_4_/N_2_ ratio of 2/1, and 36,000 mLg^−1^h^−1^ GHSV).

Table S1 catalytic activity of iron‐based catalyst (20 wt.% Fe supported on different metal oxides (Al_2_O_3_, ZrO_2_ and SiO_2_) for methane decomposition reaction. The data show that the catalytic activity of Fe‐Al is improved compared to the other supports. The initial conversions of CH_4_ and hydrogen are 69.4% and 67.1%, respectively (Figure 3). However, the CH_4_ conversion and hydrogen yields drop to 19.5% and 18%, respectively, at about 120 min of operation, indicating significant catalyst degradation. In contrast, the 20Fe/ZrO_2_ and 20Fe/SiO_2_ catalysts provided low conversion and yield values, indicating unsatisfactory catalytic efficiency. The results are probably due to the BET surface area; the 20Fe/Al_2_O_3_ has the highest surface area of 160 m^2^/g, while the other supports have considerably lower values, the 20Fe/SiO_2_ catalyst reaching 6 m^2^/g. The supports 20Fe/ZrO_2_ and 20Fe/SiO_2_ show poor performance, indicating that, in their present form, they are not suitable for efficient methane decomposition under the conditions tested. Taking the obvious advantage of Al_2_O_3_, the following researches are only focused on improving the efficacy of the 20Fe/Al_2_O_3_ catalyst. Special attention is given to modifying the alumina support to reduce the above deactivation and improve its long‐term stability. The Al_2_O_3_ is modified with 10 wt.% CeO_2_, SiO_2_, Y_2_O_3_, and/or ZrO_2_.

### 3.2 Fresh Catalyst Characterization

Figure S1 displays the images of the different support types used for the first stage. Figure S1A shows a dense, irregular agglomerate of nanoparticles with a highly textured and rough surface structure, while Figure S1B displays a more uniform, granular or spherical‐like clusters closely packed together. On the other hand, Figure S1C reveals a distinct layout containing flatter, flake‐like base structures with smaller clusters of nanoparticles.

**FIGURE 4:**
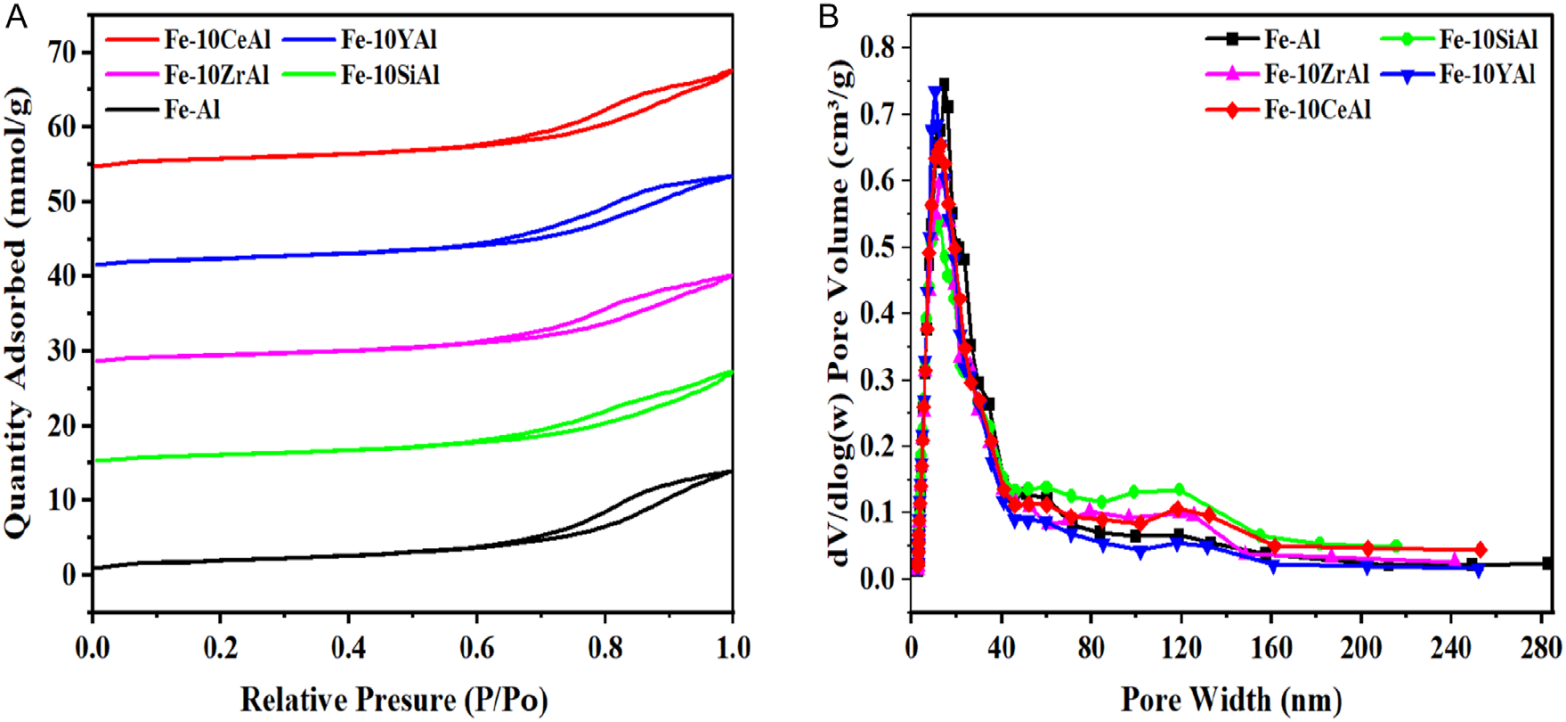
The fresh catalysts (A) BET‐N_2_ adsorption–desorption isotherms and (B) BJH distribution curves.

Table S2 summarizes the N_2_ adsorption–desorption isotherms, which are used to determine the textural characteristics of the catalysts with modified Al_2_O_3_ supports. The incorporation of either CeO_2_, SiO_2_, or ZrO_2_ into alumina resulted in a reduction of surface area from 160 m^2^/g for 20Fe/Al_2_O_3_ to 157 m^2^/g for Fe/(CeO_2_ + Al_2_O_3_), and 150 m^2^/g for both Fe/(SiO_2_+Al_2_O_3_) and Fe/(ZrO_2_+Al_2_O_3_). In contrast, with the Y_2_O_3_ incorporation, the surface area undergoes an increase (164 m^2^/g). Modified‐alumina‐supported catalysts exhibited a decrease in pore volume and pore size. The N_2_ adsorption–desorption isotherms and the catalysts’ pore size distribution are displayed in Figure 4. According to the IUPAC classification, with an H3 type hysteresis loop, all of the catalysts in Figure 4a are Type IV, demonstrating the catalysts’ mesoporous nature with slit‐shaped pores [35]. The predominant portion of the pore volume is located within the mesoporous range (2–40 nm), indicating that the majority of the pores are mesoporous, as illustrated in Figure 4b. However, the average pore size falls in the 10.4–11.3 nm range (Table S2).

**FIGURE 5:**
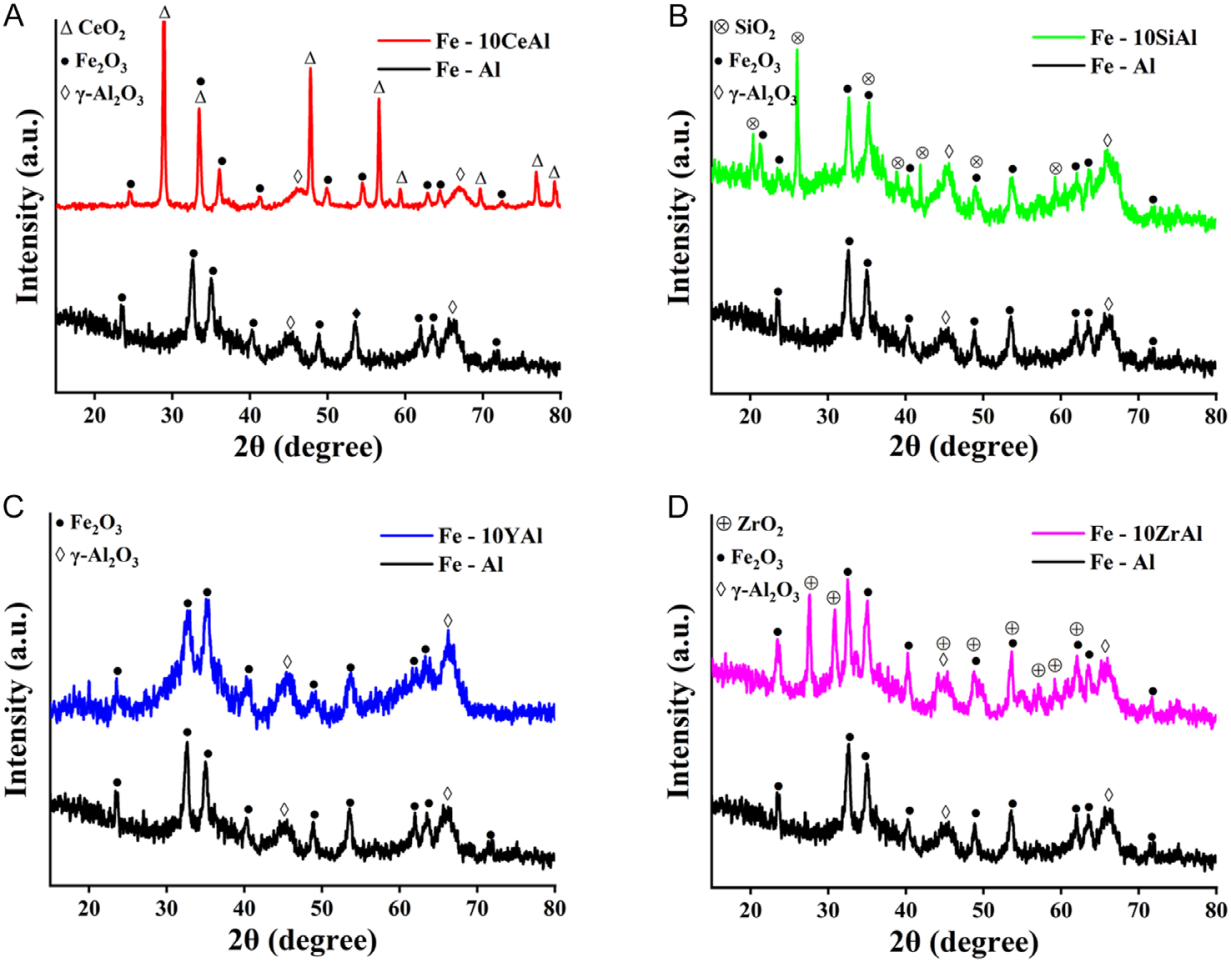
XRD profiles of modified Fe‐Al fresh catalysts, (A) with CeO_2_, (B) with SiO_2_, (C) with Y_2_O_3_, and (D) with ZrO_2_. Data profiles were subjected to standard signal smoothing to enhance visual clarity.

The fresh catalysts were further characterized by powder XRD analysis and pattern identification. As shown in Figure 5, the XRD patterns compare the Fe‐Al catalyst with different metal oxide additives (CeO_2_, SiO_2_, Y_2_O_3_, and ZrO_2_). All the samples exhibited both γ‐Al_2_O_3_ and Fe_2_O_3_ phases. The following broad peaks, located at around 2*θ* = 45.5and 66.3°, are typical of the γ‐Al_2_O_3_ (JCPDS: 96‐101−0462) [36], whereas the peaks identified at around the 2*θ* angles of 23.8°, 32.9°, 35.3°, 40.5°, 49.2°, 53.8°, 62.2°, and 63.8°could be ascribed to hematite (Fe_2_O_3_) which can match well with (JCPDS 96‐210−8029) [37]. In Fe‐10CeAl, the incorporation of CeO_2_ alongside alumina results in sharp peaks, indicating increased crystallinity compared to Fe‐Al. The Fe_2_O_3_ peaks shift slightly to higher angles due to increased crystallinity. Over this catalyst, the CeO_2_ diffraction peaks are also identified at 2*θ* = 28.9°, 33.4°, 47.8°, 56.6°, 59.3°, 69.6°, 76.8°, and 79.2°; (JCPDS 96‐900−9009). The Fe‐10SiAl catalyst has diffraction peaks at 2*θ *= 20.3°, 26°, 35.2°, 38.8°, 41.9°, 49°, and 59.2°; (JCPDS 96‐153−5067) which were assigned to SiO_2_ phase. In Fe‐10ZrAl, diffraction peaks at the 2*θ* angles of 27.6°, 30.8°, 44.9°, 48.7°, 53.62°, 57°, 59.1°, and 62°; (JCPDS 96‐810‐4265) correspond to the ZrO_2_ phase. Notably, the crystalline peak for Y_2_O_3_ is not observed over Fe‐10YAl, but the rest of the catalysts show crystalline peaks for their relevant modifiers (CeO_2_, ZrO_2_, SiO_2_). It indicates that modifiers such as Y_2_O_3_ are highly dispersed over γ‐alumina support. This behavior has been reported in the literature [38].

From the XRD patterns of Fe‐Al, Fe‐10SiAl, Fe‐10ZrAl, Fe‐10YAl, and Fe‐10CeAl catalysts, the Fe_2_O_3_ crystal sizes were calculated using the Scherrer equation and the most intense peak. The crystallite size of Fe‐Al slightly increased upon incorporation of SiO_2_ and ZrO_2_ in Fe‐Al, from 12.7 to 14.9 and 13.8 nm, respectively. Conversely, the incorporation of Y_2_O_3_ significantly reduced the crystallite size to 7.2 nm, providing supporting evidence for improved dispersion of iron species. The largest crystallite size was obtained for the CeO_2_‐modified sample, reaching 21.7 nm.

**FIGURE 6:**
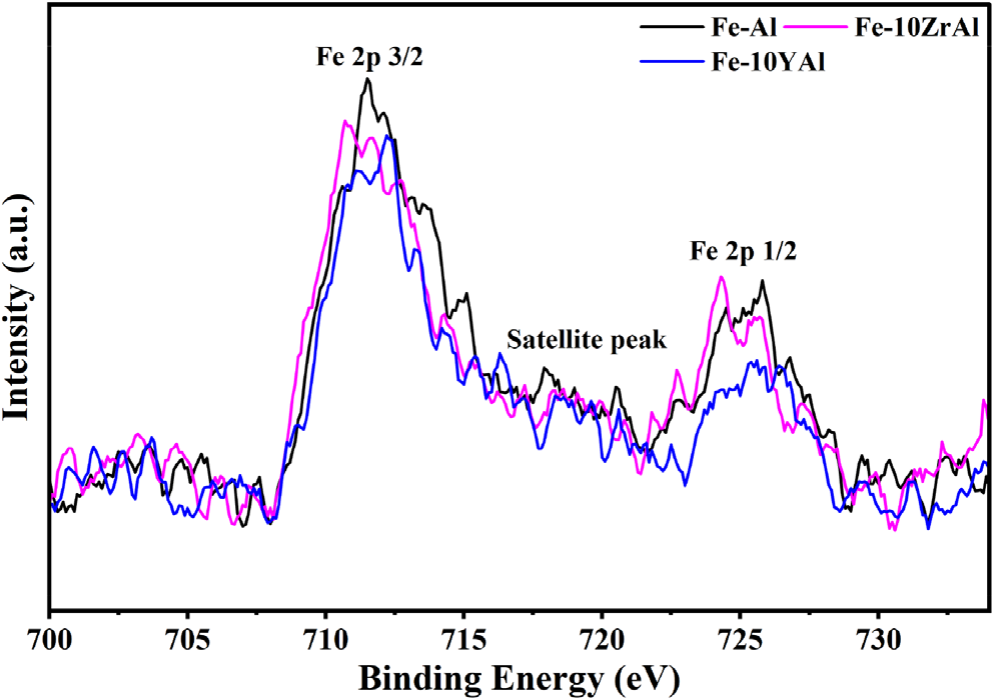
Fe 2p XPS spectra of fresh Fe–Al, Fe–10ZrAl, and Fe–10YAl catalysts.

The spectra for the selected fresh Fe‐Al and Fe catalysts supported on ZrO_2_‐ and Y_2_O_3_‐modified Al_2_O_3_ supports were extracted from XPS analysis. The binding energy region for Fe 2p is presented in Figure 6. All catalysts exhibited two main peaks at ∼710.7–711.5 and ∼724–725.5 eV, corresponding to Fe 2p_3/2_ and Fe 2p_1/2_, respectively, with a spin–orbit splitting (Δ) of ≈14 eV, indicating that Fe is present in the Fe^3+^ oxidation state [39, 40, 41]. The Fe 2p _3/2_ peak also shows a satellite feature at ∼718–719 eV. This is consistent with previous studies, where the satellite peak for Fe^3+^ appears approximately 8–8.5 eV above the main Fe 2p_3/2_ peak [42, 43, 44].

**FIGURE 7:**
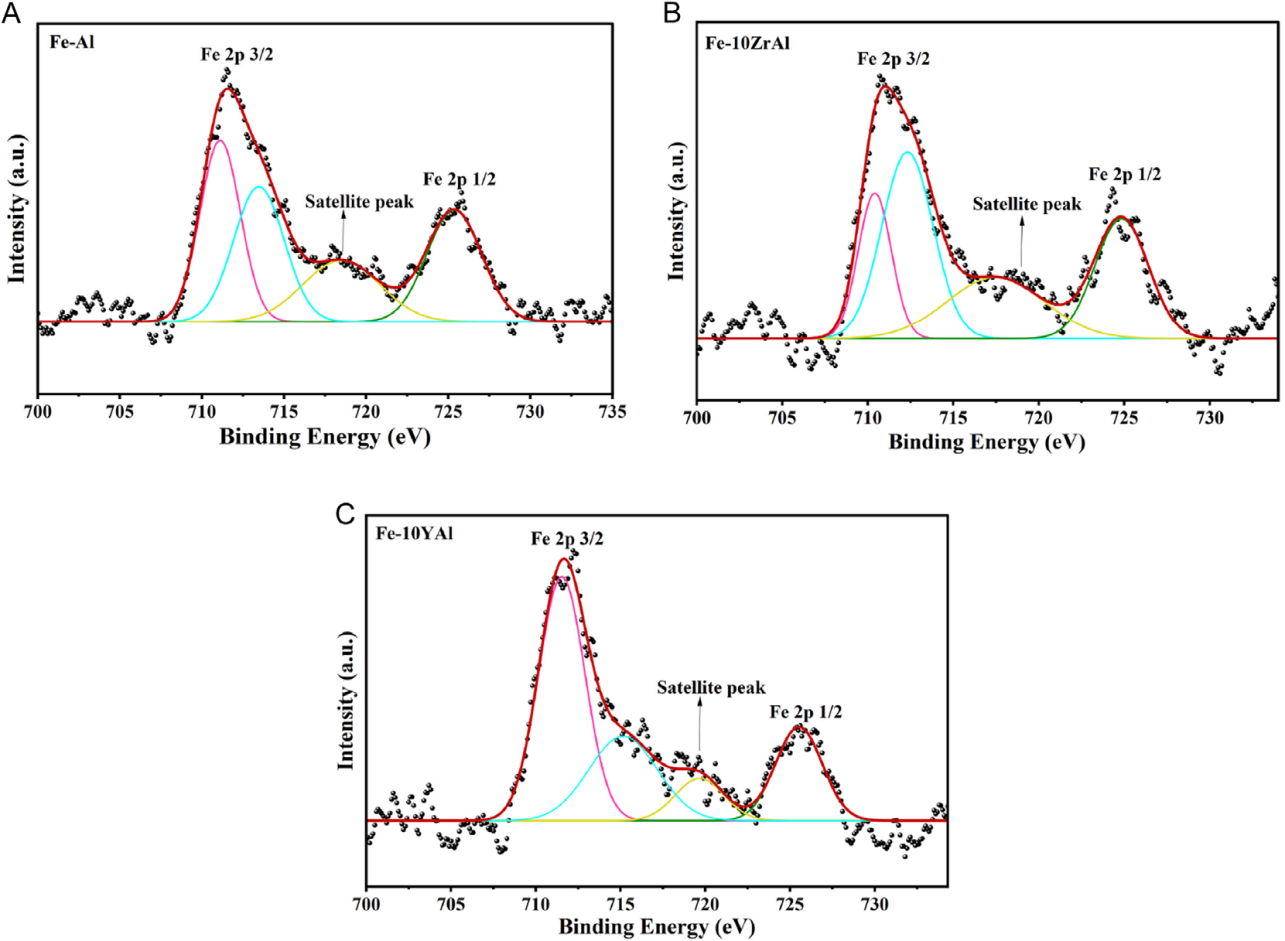
Deconvoluted Fe 2p XPS spectra of fresh Fe‐Al (A), Fe‐10ZrAl (B), and Fe‐10YAl (C) catalysts.

The Fe 2p spectra were deconvoluted using Gaussian fitting (Figure 7). The Fe 2p _3/2_ peak was resolved into two components located at ∼710–711 and ∼712–715 eV (Table 1), which indicate the presence of Fe–O bonds [45]. Furthermore, a slight chemical shift was observed depending on the support modification. For the ZrO_2_‐modified Al_2_O_3_ catalyst, the Fe 2p _3/2_ peak shifted to lower binding energy, whereas the Y_2_O_3_‐modified Al_2_O_3_ catalyst showed a slight shift toward higher binding energy. It indicates the growing electron density about Fe over ZrO_2_‐modified Al_2_O_3_ catalyst and electron deficit in the vicinity of Fe over Y_2_O_3_‐modified Al_2_O_3_ catalyst. This higher electron density about Fe can facilitate electron transfer to the antibonding orbitals of adsorbed methane, thereby promoting C–H bond activation and enhancing methane decomposition, similar to the effects observed for La_2_O_3_‐promoted Fe catalysts [46].

**TABLE 1:** XPS Fe 2p binding energies and satellite peaks for Fe catalysts on different modified alumina supports (eV).

Catalyst	Binding energy, eV			
	**Fe 2p**_**3/2**_		Satellite	**Fe 2p**_**1/2**_
Fe‐Al	711.1	713.5	718.5	725.4
Fe‐10ZrAl	710.4	712.3	717.5	724.3
Fe‐10YAl	711.5	715.2	719.7	725.5

**FIGURE 8:**
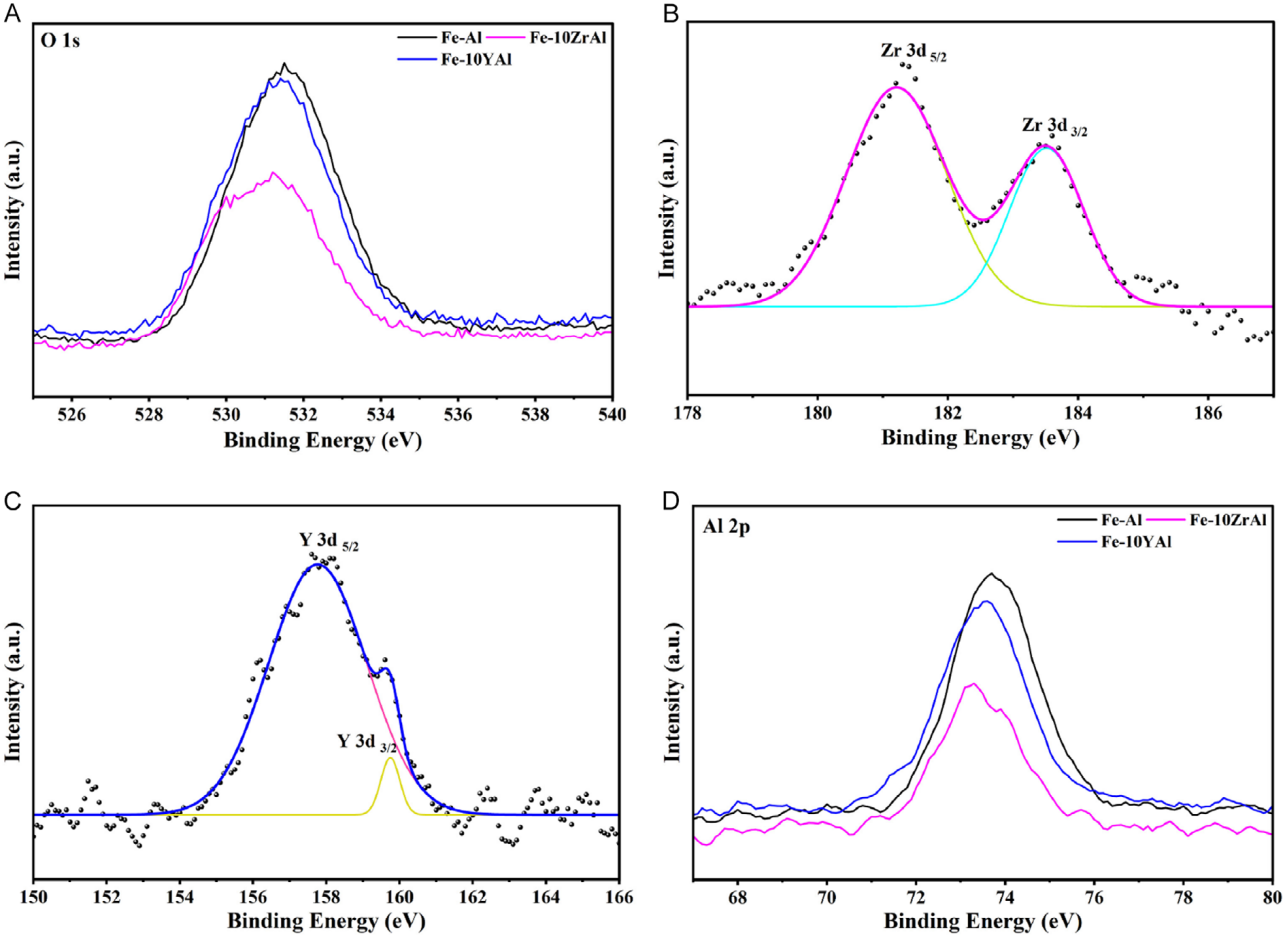
XPS spectra of catalysts (A) O 1s spectra of FeAl, Fe‐10YAl, and Fe‐10ZrAl catalysts; (B) Zr (3d) spectra of Fe‐10ZrAl; (C) Y (3d) spectra of Fe‐10YAl; (D) Al (2p) spectra of FeAl, Fe‐10YAl, and Fe‐10ZrAl catalysts.

Figure 8A shows the comparative O 1s XPS spectra of the catalysts, illustrating overlapping contributions from the lattice oxygen of metal oxides and adsorbed oxygen species [47]. The spectra reveal a slight shift toward lower binding energies for Fe‐10ZrAl and Fe‐10YAl compared with Fe‐Al. The shift indicates an increased covalent character of the M–O–M′ bonds formed upon the incorporation of ZrO_2_ and Y_2_O_3_ into the alumina support [48].

The Zr 3d XPS spectrum (Figure 8B) was deconvoluted into two peaks at 181.2 eV (Zr 3d _5__/2_) and 183.5 eV (Zr 3d _3/2_), with a splitting of ∼2.4 eV, which are in good agreement with the reported binding energy values for Zr 3d doublets of zirconia in the literature [49]. The binding energy of Zr 3d _5/2_ in ZrO_2_ is typically reported in the range of 182.2–182.4 eV, while lower values have been attributed to oxygen vacancies in the crystalline structure of the ZrO_2_ [50, 51], which is consistent with the slightly lower binding energy observed in the present study. This phenomenon has been observed in composite Fe_2_O_3_ and ZrO_2_/Al_2_O_3_ catalysts [50], as well as annealed TiO_2_ films [52]. The Y 3d XPS spectrum (Figure 8C) was deconvoluted into two peaks at approximately 157.8 and 159.7 eV, corresponding to the Y 3d _5/2_ and Y 3d _3/2_ spin–orbit components, respectively, with a splitting of about 2.0 eV, characteristic of Y^3+^ species. These binding energies are higher than those reported for pure Y_2_O_3_ (156.4 and 158.5 eV), likely due to interaction between Y and Al species, which alters the local chemical environment of yttrium, [53, 54]. The observed splitting is consistent with values reported for Y^3+^ in other yttrium‐containing compounds [55]. The Al 2p spectra (Figure 8D) of the Fe‐10ZrAl and Fe‐10YAl catalysts show a dominant peak at around 73.3–73.7 eV, corresponding to Al^3+^ in Al_2_O_3_. Slight shifts in the binding energy among the samples may be attributed to interactions between Al_2_O_3_ and the added oxides (ZrO_2_ and Y_2_O_3_). Incorporation of the dual metal oxide supports reduces the Al^3+^ peak intensity, likely due to partial coverage of the Al_2_O_3_ surface by other cationic species [47].

**FIGURE 9:**
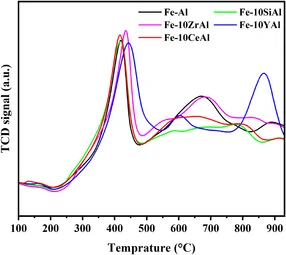
H_2_‐TPR patterns for fresh supported catalysts.

Figure 9 shows the H_2_‐TPR spectra of the Fe‐Al catalyst and Fe catalysts supported on modified alumina. To quantitatively evaluate the reducibility behavior, the hydrogen consumption values and reduction percentages are summarized in Table 2. Generally, all catalysts exhibit a distinct initial reduction peak, followed by a broad and overlapping reduction zone at higher temperatures, with a third characteristic high‐temperature reduction peak appearing in some samples. This suggests they undergo several sequential reduction stages, a characteristic commonly observed among iron‐based catalysts, which, as previously mentioned, can form various oxide phases. The observed peaks indicate the stepwise reduction of iron (III) oxide to elemental iron, in the sequence: Fe_2_O_3_ → Fe_3_O_4_ → FeO → Fe, where each stage corresponds to a decrease in iron's oxidation state. The sharp peak observed between 300 °C and 500 °C indicates the transformation of Fe_2_O_3_ → Fe_3_O_4_ [56] (shown by T1). Concurrently, the broader and overlapping reduction peaks identified within the 500 °C to around 800 °C range correspond to the overlapping reduction of Fe_3_O_4_ to FeO (Wüstite) phase and the subsequent reduction of FeO to metallic Fe [28, 56] (shown by T2). The third region (high‐temperature region, T3) is the last reduction of highly dispersed FeO to active metallic iron (Fe^0^), which is strongly dependent on the metal–support interaction. The analysis of the different profiles shows that shifting to higher temperatures clearly demonstrates how modifications of the support affect the extent of metal–support interaction and the overall reducibility of the active phases.

**TABLE 2:** Hydrogen consumption of fresh catalyst.

Catalyst	**Amount (cm^3^/g STP)** a **temperature range, °C**			**Total H**_**2**_ **(cm^3^/g STP)** a	**(Reduction)** b **%**
	≤500	500–800	>800		
Fe‐Al	14.4	10.1	0.9	25.4	21.10
Fe‐10SiAl	17.56	1.54	0.88	19.98	16.59
Fe‐10ZrAl	16.10	10.2	0.61	26.91	22.15
Fe‐10YAl	18.27	1.64	11.54	31.45	26.12
Fe‐10CeAl	17.23	4.96	1.76	23.95	19.89
Fe_2_O_3_	41.2	4.66	27.62	73.48	61.03

a Evaluated from TPR.

b Definition of % reduction = Experimental H_2_‐consumption/Theoretical H_2_‐consumption (120.4 cm^3^/g STP).

A shift in the reduction temperature toward a higher value (above 800 °C) accompanied by a high‐intensity reduction peak for the Fe‐10YAl catalyst indicates a stronger metal–support interaction. Fe‐10YAl exhibited the highest total hydrogen consumption, reaching 31.45 cm^3^/g STP with a reduction percentage of 26.12% compared with Fe‐Al, which showed 25.4 cm^3^/g STP and a reduction percentage of 21.10% reduction. The high‐temperature reduction behavior of Fe‐10YAl, extending up to and beyond 800 °C, provides essential information regarding the strength of the metal–support interactions. This strong homogeneous interfacial bonding restricts the mobility of the iron species preventing thermal sintering and maintaining high dispersion of the active phases under demanding high‐temperature reaction conditions. Again, the high‐temperature reduction peaks indicate a strong interfacial bonding, which limits the reducibility of iron species despite the initial hydrogen treatment being performed at 700 °C. However, the amount of hydrogen consumed in the 500–800 °C range was lower with Fe‐10YAl compared to Fe‐Al. This suggests that, for the catalytic methane breakdown reaction at 800 °C, very few active sites are available over the Fe‐10YAl catalyst. Within the working temperature range, among the modified catalysts, the ZrO_2_‐modified catalyst consumed the highest amount of hydrogen, with improved reducibility observed during reduction. In contrast, Fe‐10CeAl exhibited 23.95 cm^3^/g STP H_2_ consumption and 19.89 % reduction. Hydrogen consumption may also arise from the reduction of the ceria modifier, and the net H_2_ consumption by iron oxide would be lower for the Fe‐10CeAl catalyst. Fe‐10SiAl catalyst had the minimum hydrogen consumption among the catalysts. After Fe‐10YAl catalyst, the Fe‐10ZrAl and Fe‐Al catalysts have prominent reduction in the high‐temperature range (>500^o^C) indicating stronger metal support interaction over Fe‐10ZrAl and Fe‐Al catalysts than Fe‐10CeAl and Fe‐10SiAl catalysts. The reduction profile of the Fe‐10ZrAl catalyst is also slightly shifted towards higher temperatures compared with that of the Fe‐Al catalyst. This indicates stronger metal–support interaction in Fe‐10ZrAl than in Fe‐Al.

The H_2_‐TPR profile of pure Fe_2_O_3_ is shown in Figure S2 for comparison with the supported catalyst samples. The reduction peak of Fe_2_O_3_ at around 399 °C is attributed to the reduction of Fe_2_O_3_ to Fe_3_O_4_, while the broad reduction peak observed from about 450 to above 800 °C corresponds to the sequential reduction process of Fe_3_O_4_ → FeO → Fe, in good agreement with previous reports [57]. Compared with the supported Fe catalysts shown in Figure 9, pure Fe_2_O_3_ exhibits a dominant sharp peak at low temperature, whereas the supported catalysts exhibit more complex reduction features at intermediate and high temperatures. This observation suggests that metal–support interactions may affect reducibility behavior.

### 3.3 Catalytic Activity Results

**FIGURE 10:**
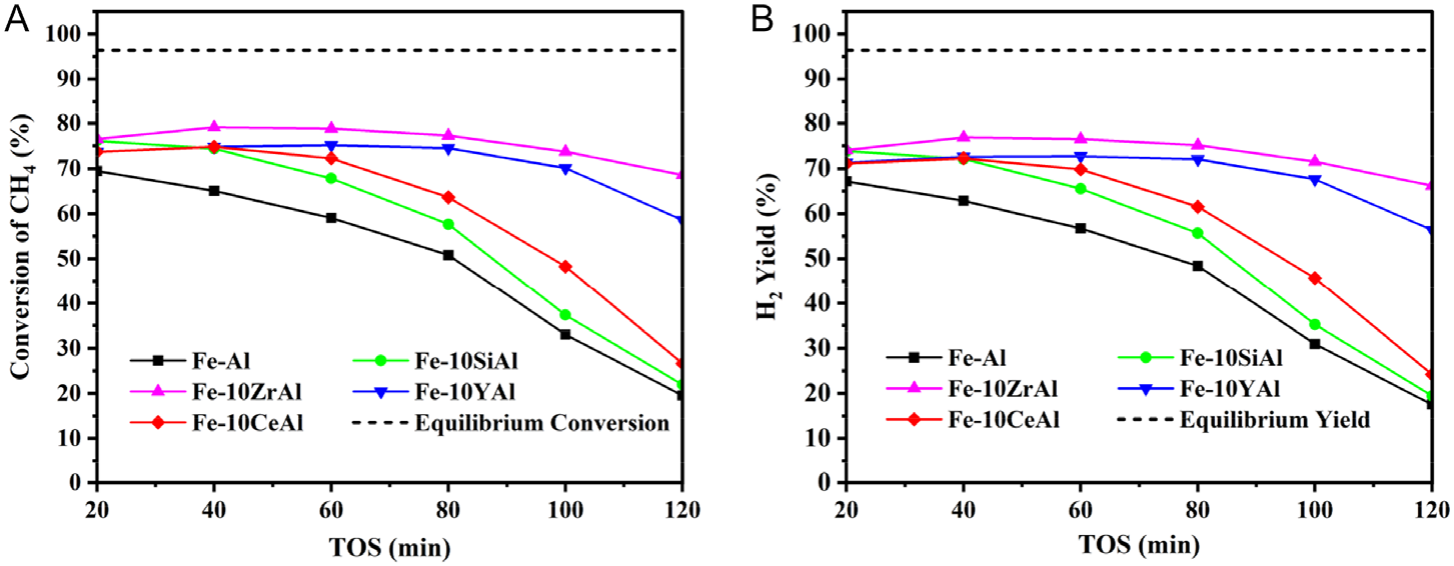
Catalytic activity results: (A) CH_4_ conversion (%), (B) H_2_ yield (%), at 800 °C and 120 min TOS, *p* = 1 atm, CH_4_:N_2_ feed ratio = 2:1, and GHSV = 36,000 mLg^−1^h^−1^.

The catalytic activity data for the methane conversion during a 120‐min time on‐stream (TOS) are shown in Figure 10. The activity patterns demonstrate that adding metal oxides to Al_2_O_3_ significantly enhances both methane conversion and hydrogen yield. The results showed that as the reaction progressed, the Fe‐10ZrAl catalyst produced the highest methane conversion with higher stability. The initial conversion rates of CH_4_ and the yield of H_2_ for the Fe‐10ZrAl commenced at approximately 76.5% and 74.1%, respectively, and subsequently declined to 68.5% and 66.2% after 120 min of on‐stream. In contrast, the Fe‐10SiAl catalyst showed an initial CH_4_ conversion of approximately 76.1%, similar to that of Fe‐10ZrAl; however, after 120 min of TOS, it rapidly deactivated, reaching a low value of 21.9%. The Fe‐10YAl and Fe‐10CeAl catalysts initially demonstrated comparable CH_4_ conversion rates and H_2_ yields, approximately 74% and 71%, respectively. However, the Fe‐10YAl catalyst demonstrated a slight increase in activity performance, maintaining relative stability for up to 80 min. Following that, a decrease in CH_4_ conversion (58.6%) and H_2_ yield (56.3%) was observed. In contrast, after 60 min, the performance of the Fe‐10CeAl catalyst drastically decreased. The CH_4_ conversion and H_2_ yield dropped to 26.6% and 24.2% at the end of 120 min, respectively.

The dashed line represents the thermodynamic equilibrium of CH_4_ conversion /H_2_ yield under the reaction conditions. CH_4_ conversion and H_2_ yield are calculated at approximately 96% under the specified reaction conditions. All catalysts studied exhibited values below the equilibrium limit. Among these catalysts, Fe‐10ZrAl exhibits the closest approach to equilibrium. Table 3 compares several Fe‐based catalysts for CH_4_ decomposition, highlighting how different metal oxide modifiers (SiO_2_, ZrO_2_, Y_2_O_3_, and CeO_2_) influence catalytic activity and carbon accumulation.

**TABLE 3:** Comparison of Fe‐based catalysts: methane conversion, H_2_ yield, surface area, weight loss, and carbon deposition.

Catalyst	**CH**_**4**_ **conversion %**		**H**_**2**_ Yield %		***S***_**BET**_ **(m** ^**2**^ **/g)** a		**Weight loss** b **, %**	gC/gFe
	initial	final	initial	final	fresh	used		
Fe‐Al	69.4	19.5	67.1	18	160	119	48.1	4.6
Fe‐10SiAl	76.1	21.9	73.8	19.4	150	119	54.1	5.9
Fe‐10ZrAl	76.5	68.5	74.1	66.2	150	113	55.1	6.4
Fe‐10YAl	73.7	58.6	71.3	56.3	164	125	61.1	7.9
Fe‐10CeAl	73.7	26.6	71.1	24.2	157	137	55.1	6.2

a *S*_BET_ (surface area).

b Evaluated from TGA.

**FIGURE 11:**
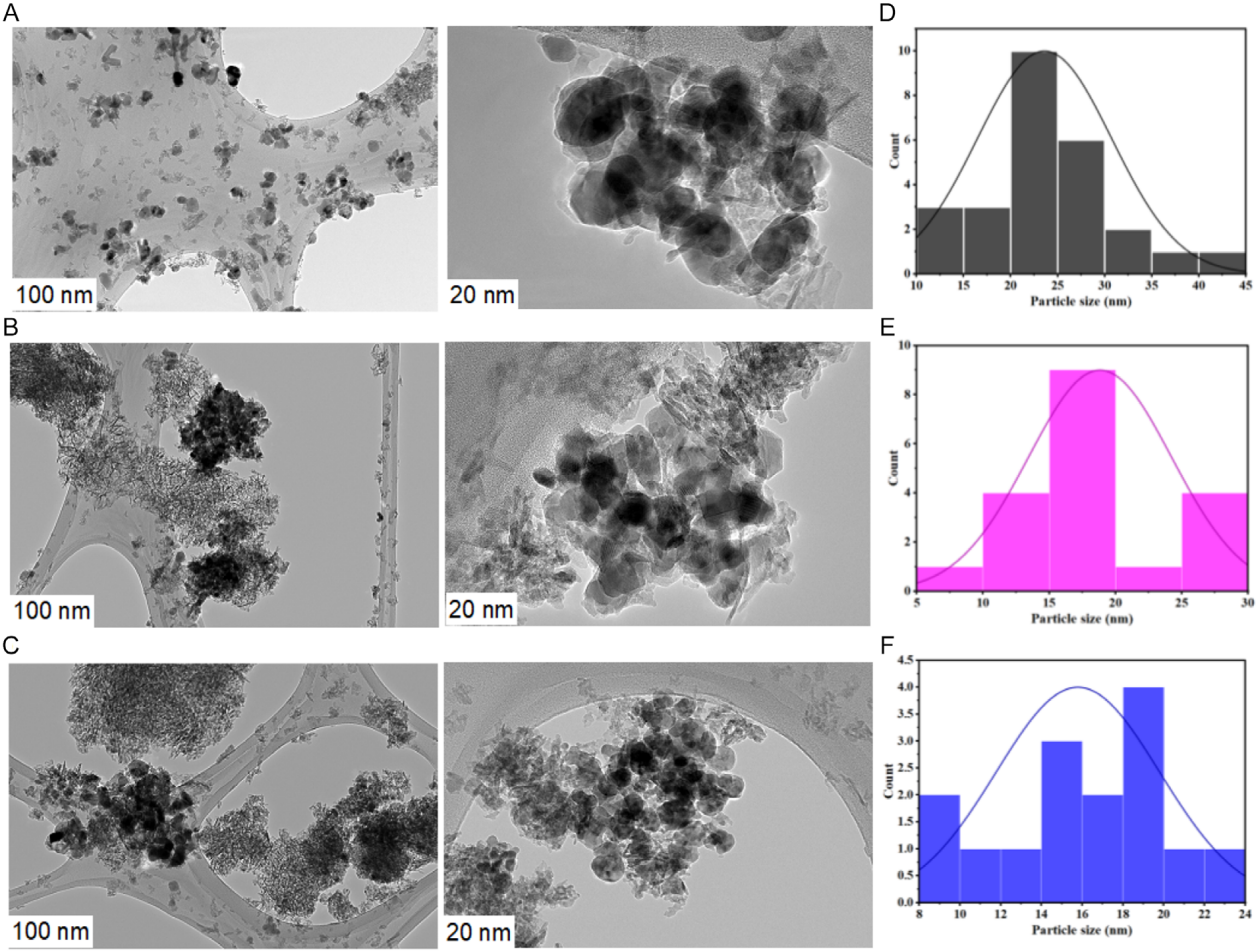
TEM image of fresh (A) Fe‐Al, (B) Fe‐ZrAl, and (C) Fe‐YAl catalysts on 100 and 20 nm scales; particle size distribution of fresh (D) Fe‐Al, (E) Fe‐10ZrAl, and (F) Fe‐10YAl samples.

Figure 11 illustrates the findings from the examination of the internal microstructure of the calcined catalysts, as assessed through TEM analysis. Figure 11A–F display the TEM images at two distinct magnifications (100 and 20 nm), along with the particle size distributions for Fe‐Al, Fe‐10ZrAl, and Fe‐10YAl, respectively. Generally, Fe species nanoparticles are visible in the TEM pictures of the supported catalysts, appearing as dark spots dispersed to varying degrees over the metal oxide supports. The Fe‐Al catalyst shows semi‐spherical Fe nanoparticles with noticeable agglomeration in certain regions of the support. Furthermore, the particle size distribution exhibits a maximum average of 23.6 nm, with sizes ranging from 13 to 43 nm. An irregular‐shaped Fe nanoparticle with an average particle size distribution of 18.8 nm is detected in the Fe‐10ZrAl catalyst, exhibiting a size range of 9–28 nm. The Fe‐10YAl displays the highest Fe dispersion compared to both Fe‐Al and Fe‐10ZrAl. Besides, this catalyst exhibited the smallest average nanoparticle size of 15.8 nm (semi‐spherical shape) and a narrower particle size distribution, with a peak in the 8–22 nm size range. The non‐uniform distribution of Fe nanoparticles over Al_2_O_3_ support can be attributed to particle migration and coalescence during calcination. In contrast, adding either ZrO_2_ or Y_2_O_3_ to alumina has enhanced the dispersion of Fe. This underscores the crucial role of oxide additives. Y_2_O_3_ offers more effective anchoring sites for Fe or facilitates stronger metal–support interactions than ZrO_2_, resulting in improved dispersion.

### 3.4 Spent Catalyst Characterization

**FIGURE 12:**
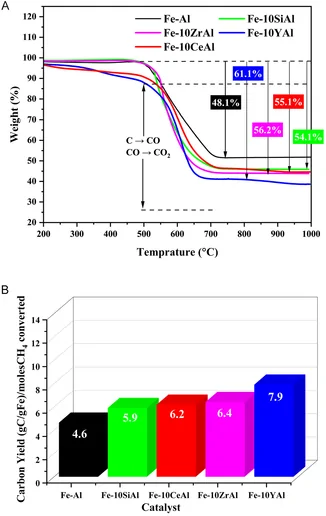
(A) TGA curves and (B) carbon yield for used catalysts after 120 min of reaction at 800 °C.

To acquire quantitative data on TGA analysis and carbon content, TGA measurements were performed on spent catalysts, as seen in Figure 12. The observed weight loss of the spent catalysts is due to the oxidation of carbon produced on the samples during the CDM process (Figure 12A). The Fe‐Al catalyst has the lowest weight loss of 48.1%, while the Fe‐10YAl exhibited the highest weight loss of 61.1%. For Fe‐Al, Fe‐10ZrAl, and Fe‐10SiAl, the weight loss followed a similar pattern, starting above 500 °C and decreasing as the temperature rose. On the other hand, the Fe‐10YAl and Fe‐10CeAl catalysts exhibited a gradual decrease in weight loss, which began at 300 °C. Amorphous carbon is typically oxidized below 450 °C [58]. All catalysts, including the original Fe‐Al catalyst and the modified Al_2_O_3_‐supported catalysts, demonstrated carbon yields consistent with their activity, except for Fe‐10ZrAl. As illustrated in Figure 12B, the carbon yield over Fe‐Al, Fe‐10Y‐Al, Fe‐10Zr‐Al, Fe‐10Ce‐Al, and Fe‐10Si‐Al is 4.6gC/gFe, 7.9 gC/gFe, 6.4 gC/gFe, 6.2 gC/gFe, and 5.9 gC/gFe, respectively. The carbon yield order across different catalysts is consistent with the weight loss (%) in the TGA analysis. The low carbon yield over Fe‐10Si‐Al and Fe‐10Ce‐Al catalysts is due to their lower catalytic activity towards CH_4_ decomposition. Thus, the higher carbon yield for the ZrO_2_‐modified Al_2_O_3_‐supported catalyst, as shown in Table 3 and Figure 12, indicates its excellent catalytic activity and improved resistance to deactivation. The improved support allows for sustained, long‐term growth of structured graphitic carbon during the time‐on‐stream test, as opposed to rapid carbon deposition that stops the reaction early on the reference Fe/Al_2_O_3_ catalyst.

**FIGURE 13:**
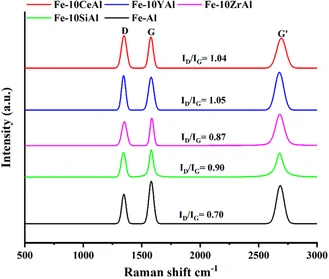
Raman spectra for the used catalysts at 800 °C.

X‐ray diffraction analysis of the spent catalysts revealed the formation of the iron carbide phase, a well‐known intermediate in the carbide cycle mechanism of methane decomposition. Due to the structural similarities and overlapping diffraction lines of Fe_3_C polymorphs (such as cementite and cohenite), these are indexed collectively as the iron carbide phase and graphite, as illustrated in Figure S3. The detection of Fe_3_C across all samples suggests that iron significantly contributes to methane decomposition, leading to the formation of carbides. The quality of carbon nanomaterials formed on the catalyst surface was assessed through spectroscopic analysis. As illustrated in Figure 13, all spent catalysts revealed three distinct Raman bands. These bands were detected at 1348, 1583, and 2690 cm^−1^, corresponding to the D, G, and G’ bands, respectively. Generally, the D band is assigned to disordered carbon or structural imperfections in the graphitic layers, while the G band corresponds to the in‐plane vibration of sp^2^‐hybridized carbon atoms in ordered graphitic layers. The G’ band results in the higher‐order two‐phonon process associated with the set of graphene layers in carbon nanomaterials [59, 60]. The *I*_D_/*I*_G_ value is usually used as an important parameter to determine the graphitization degree of the carbon. The decrease in the *I*_D_/*I*_G_ value indicates an increase in the crystalline order of the carbon materials. Fe‐Al, Fe‐10SiAl, and Fe‐10ZrAl exhibited ratios of 0.70, 0.90, and 0.87, respectively. In contrast, Fe‐10YAl and Fe‐10CeAl showed significantly higher ratios, 1.05 and 1.04, suggesting a greater prevalence of disordered structures on these catalysts. The lowest *I*_D_/*I*_G_ over‐spent Fe‐Al catalyst indicates that the degree of graphitization of carbon is highest over this catalyst. The Raman spectroscopy analysis's results aligned with the TGA investigations’ conclusions. The degree of graphitization correlates with the carbon oxidation temperature observed in the TGA analysis. Specifically, the CeO_2_‐ and Y_2_O_3_‐modified catalysts demonstrated carbon oxidation at lower temperatures, indicating the formation of amorphous carbon species.

**FIGURE 14:**
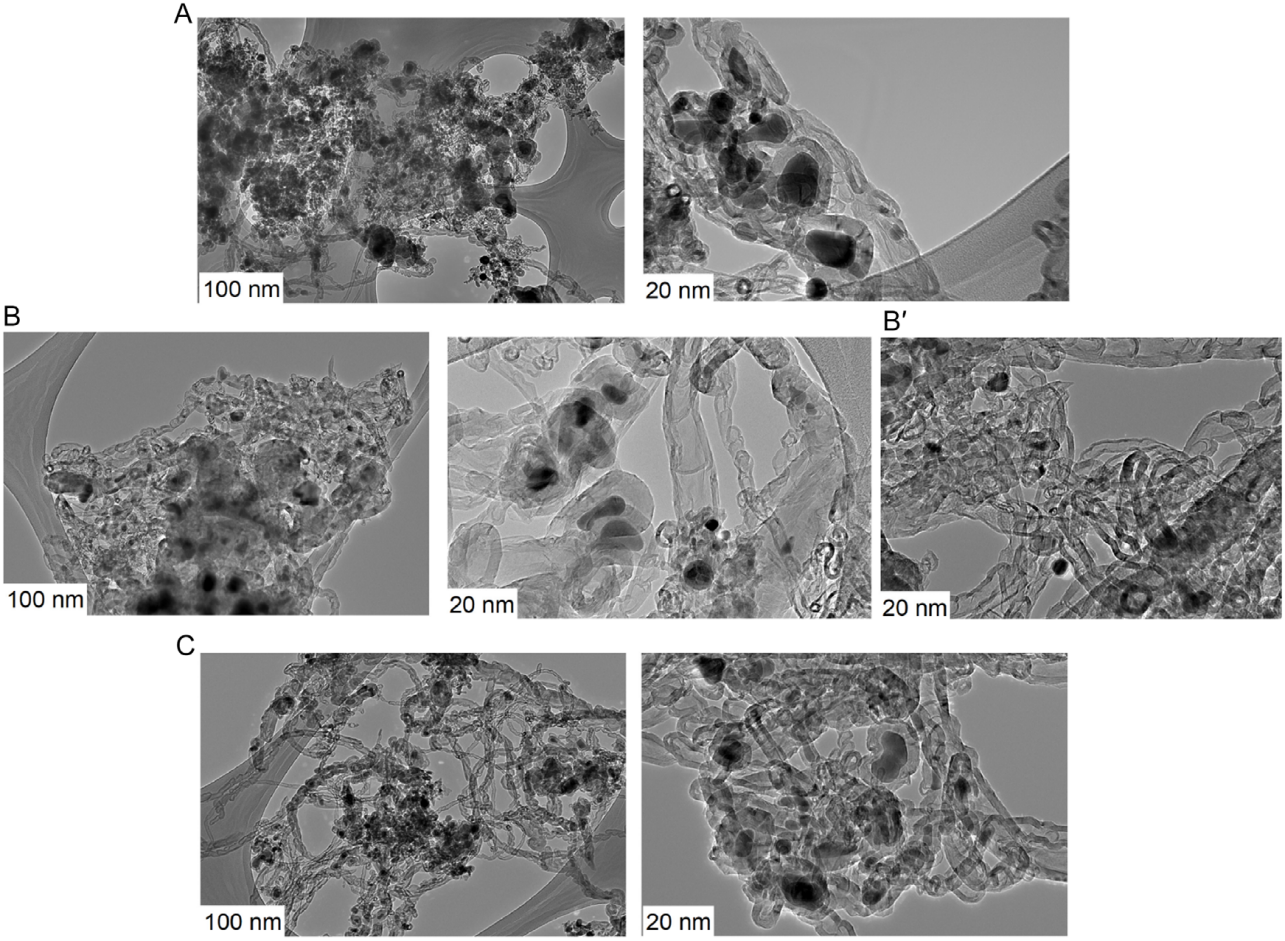
TEM image of spent (A) Fe‐Al, (B)10ZrAl, and (C) Fe‐10YAl catalysts on 100 and 20 nm scales; higher regular morphological carbon of spent Fe‐10ZrAl catalyst (B′).

TEM micrographs of the spent Fe‐Al, Fe‐10ZrAl, and Fe‐10YAl catalysts are provided in Figure 14 to show the morphology of the carbon formed. Multi‐walled carbon nanotubes (MWCNTs), having a hollow tubular carbon structure, grew as a result of the catalysts. The average nanotube diameters for the Fe‐Al, Fe‐10ZrAl, and Fe‐10YAl catalysts were 29, 20, and 23 nm, respectively. The Fe‐10ZrAl catalyst demonstrates the narrowest distribution (Figure S4), signifying improved nanoparticle stability and regulated MWCNTs growth (Figure 14B′). This is likely attributed to ZrO_2_'s ability to prevent sintering of Fe nanoparticles. Moreover, most of the Fe nanoparticles remained adhered to the substrate, suggesting that MWCNT growth on this catalyst is primarily consistent with a base‐growth process. A tip‐growth mechanism is suggested for Fe‐10YAl, which carries active metal particles at CNT tips. In the case of the Fe‐Al catalyst, metal particles (dark spots) at the tip and within the carbon nanostructures were observed, suggesting both tip‐ and base‐growth mechanisms [61]. From the TEM micrographs, black, densely aggregated nanoparticles (associated with the iron/iron carbide phases identified by XRD) are seen at the junctions and tips of the short, interconnecting CNTs. The distribution shows a complex growth behavior, in which the strength of the local metal–support interaction may influence the carbon nanotube growth pathway. It is interesting to note that in the case of Fe‐Al and Fe‐10YAl catalysts, Fe particles were encapsulated into CNTs. Although this phenomenon was observed in Fe‐10ZrAl, it occurred in a lower form. This observation could explain the drop in these catalysts for methane decomposition. Fe‐Al (Figure 14A) shows that particles have been agglomerated into larger particles, which can also be seen on Fe‐10YAl (Figure 14C) and Fe‐10ZrAl (Figure 14B). Notably, the latter has a lower tendency to agglomerate, indicating enhancement in sintering‐resistance. The ZrO_2_ can inhibit the metal sintering, reduce carbon deposition, and improve the thermal stability of the catalyst [32, 62]. Therefore, ZrO_2_‐modified Al_2_O_3_ is suitable for achieving a good balance between catalytic activities and sintering inhibition. It is noteworthy that adding ZrO_2_ and Y_2_O_3_ to alumina improved the uniformity of CNTs produced. It can be challenging to precisely measure nanotube length due to their tendency to aggregate and tangle.

## 4 Discussion

Among mono‐metal oxide supports, the 20Fe/Al_2_O_3_ catalyst (Fe‐Al) exhibits the largest surface area of 160 m^2^/g, and other supports (20Fe/SiO_2_ and 20Fe/ZrO_2_) demonstrate significantly lower values (below 10 m^2^/g). An adequate surface area is required to provide an appropriate number of active metal sites for the reaction. Consequently, the 20Fe/SiO_2_ and 20Fe/ZrO_2_ catalysts were deemed unsuitable for further modification in this study due to their limited textural properties. The Fe‐Al catalyst achieved initial conversions up to 69% and 67% H_2_ yield, though it suffered from rapid deactivation over time on stream due to severe iron sintering and graphitic carbon deposition. Therefore, the Fe‐Al catalyst was further modified with SiO_2_, ZrO_2_, Y_2_O_3_, and CeO_2_ to improve its catalytic performance and stability. The resulting Al_2_O_3_‐supported catalysts (Fe‐10SiAl, Fe‐10ZrAl, Fe‐10YAl, and Fe‐10CeAl) exhibited mesoporous structures with average pore sizes ranging from 10.4 to 11.4 nm. The quantitative data on crystalline size, specific surface area, and the relative abundance of reducible species (based on the intermediate region of H_2_‐TPR) for the Fe‐Al catalyst and its modified counterparts are shown in Table S3. Metal oxide‐modified alumina‐supported Fe catalysts (Fe‐10MAl; M = Si, Zr, Y, Ce) acquire adequate surface area (≥150 m^2^/g), larger Fe crystallite sizes than the Fe‐Al catalyst with the exception of that of Fe‐10YAl, together with reduced graphitic carbon deposition (Figure 13). Since the specific surface areas of the modified catalysts were comparable, the relative abundance of reducible species under the reaction conditions becomes the key factor governing their catalytic performance. Among them, Fe‐10ZrAl exhibited the highest abundance of reducible species, followed by Fe‐10YAl, whereas Fe‐10CeAl and Fe‐10SiAl showed lower abundances (Table S3). The presence of the iron carbide (Fe_3_C) phase on spent catalysts indicates that the reaction mechanism for CH_4_ decomposition and carbon precipitation in the current catalyst system is based on Fe_3_C (the detailed mechanism is discussed below). Overall, Fe‐10ZrAl outperformed other catalysts in the methane decomposition reaction, and Fe‐10SiAl had the worst performance. Fe‐10ZrAl catalyst shows ∼74‐69% CH_4_ conversion and ∼77‐66% H_2_ yield during 120 min of reaction. The Fe‐10ZrAl catalyst also exhibits the narrowest distribution of MWCNTs (average diameter of 20 nm). The Fe‐10YAl and Fe‐10SiAl catalysts had adequate surface area as well as comparable abundance of reducible species with 800°C reaction temperature. Then, the dispersion of active sites decides catalytic performance. Fe‐10YAl exhibited a smaller Fe crystallite size (7.2 nm) than Fe‐10SiAl, which may have contributed to its improved catalytic stability. Consequently, catalytic activity drops faster over Fe‐10SiAl than Fe‐10YAl. At the end of 120 min, CH_4_ conversion and H_2_ yield reach ∼59% and 56%, respectively, over the Fe‐10YAl catalyst. The catalytic activity of Fe‐10SiAl catalysts is inferior, and minimum CH_4_ conversion (22%) and minimum H_2_‐yield (19%) are retained at the end of 120 min. The catalytic behavior of the CeO_2_‐modified catalyst differs from that of the other modified catalysts and therefore warrants separate discussion. Ceria may affect the reducibility of Fe species and influence their redox behavior. Consequently, the net abundance of reducible species over Fe‐10CeAl is lower than that over Fe‐10YAl. As a result, the catalytic activity of Fe‐10CeAl is inferior to that of Fe‐10YAl. Performance comparisons of the designed catalysts with those reported in the literature are listed in Table S4. The Fe‐10YAl and Fe‐10ZrAl catalysts have very competitive performance. Although some reported catalyst systems, i.e., 20% Fe/ La_2_O_3_ + ZrO_2_, 15%Fe/CeZrO_2,_ and 20%Fe/CeO_2_‐Al_2_O_3_, show an initial higher CH_4_ conversion (>80%), their catalytic activities declined rapidly with time on stream. However, Fe‐10YAl and Fe‐10ZrAl catalysts achieve 73.7%–58.6% CH_4_ conversions and 76.5%–68.5% CH_4_ conversions during 120 min time on stream, respectively. Overall, Fe‐10YAl and Fe‐10ZrAl catalysts exhibit consistent, high catalytic activity towards the CO_*x*_‐free methane decomposition reaction.

**FIGURE 15:**
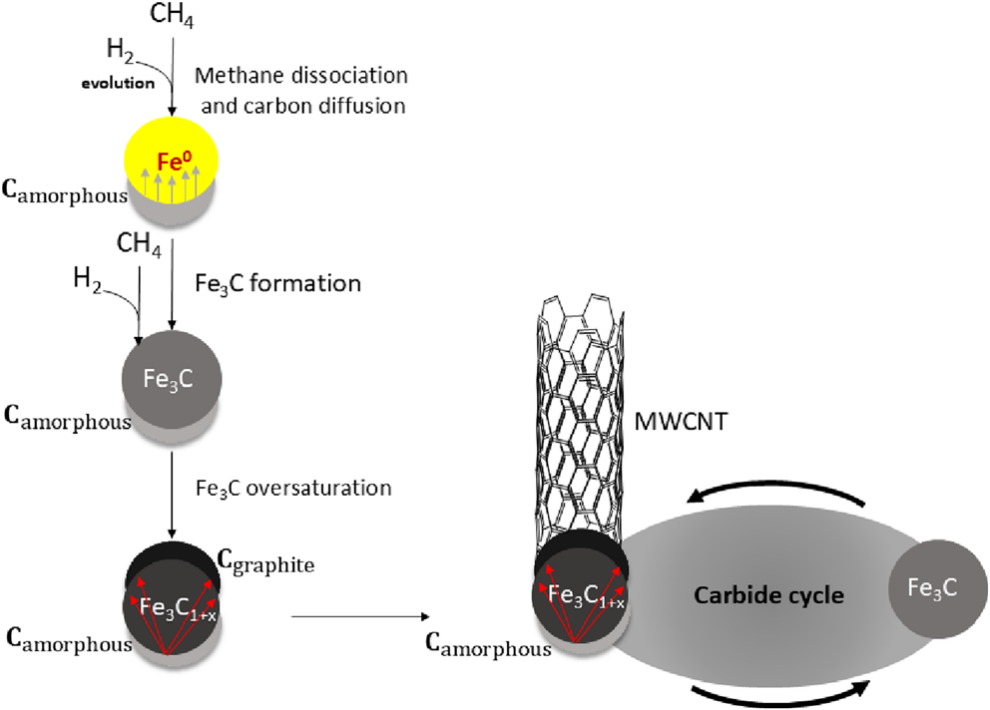
Schematic of the mechanism of methane decomposition over the Fe catalyst and carbide cycle. Content modified based on the conceptual framework reported in ref. [25].

Here, the detailed Fe_3_C‐based reaction mechanism needs to be discussed. The catalytic decomposition of methane CDM over iron‐based catalysts is initiated by the dissociative adsorption (chemisorbed) of CH_4_ molecules on Fe^0^ active sites. In this step, the C·H bonds in methane are progressively cleaved, producing H_2_ and solid carbon [63]. The amorphous carbon formed may either accumulate on and encapsulate the catalyst surface, shielding it from reactants and leading to catalyst deterioration, or diffuse into Fe^0^ particles, forming iron carbide (Fe_3_C). With continued exposure to CH_4_, Fe_3_C may become oversaturated (Fe_3_C_1+*x*_), illustrated in Figure 15, triggering the precipitation of graphitic carbon, such as CNTs, while regenerating Fe_3_C for continued activity. This mechanism, known as the carbide cycle, is supported by our XRD and TEM findings and aligns with a previous study [25], which demonstrated that Fe_3_C functions as a carbon‐transfer intermediate and facilitates the nucleation of graphitic carbon. Based on the experimental observations and the sequence of reactions reported in [25], the following reactions under Equations (II)–(V) can occur:

(II) $${\mathrm{CH}}_{4(g)}\;\;\overset{{\mathrm{Fe}}^{o}}{\to }\;C_{\left(s\right)}+2H_{2(g)}$$

(III) $${3{\mathrm{Fe}}^{0}}_{\left(s\right)}+C_{\left(s\right)}\;\to \;{\mathrm{Fe}}_{3}C_{\left(s\right)}$$

(IV) $$x{\mathrm{CH}}_{4(g)}+{\mathrm{Fe}}_{3}C_{\left(s\right)}\;\to \;{\mathrm{Fe}}_{3}C_{1+x}(s)+2xH_{2(g)}$$

(V) $${\mathrm{Fe}}_{3}C_{1+x}\;\to \;{\mathrm{Fe}}_{3}C+\text{x}C(\text{carbon nanotube})$$

This pathway is commonly referred to as a carbide cycle.

The Fe_3_C‐driven route in this study is robustly verified by the experimental findings. The XRD patterns of the spent catalysts exhibited distinct diffraction peaks corresponding to Fe_3_C (cementite), signifying its role as a critical intermediate in the methane decomposition process. This phase likely functions as a carbon reservoir, facilitating the transformation of amorphous carbon into more structured forms such as graphite or CNTs. TEM analysis verified the formation of well‐structured MWCNTs over Fe‐10ZrAl and Fe‐10YAl, supporting the role of Fe_3_C as an active intermediate in carbon growth. The findings indicate that, while all catalysts employ the same fundamental Fe_3_C‐mediated mechanism, the support composition is pivotal in shaping the carbon structure, catalyst stability, and the overall efficiency of CDM.

## 5 Regeneration Study and Raman Characterization of the Spent Catalysts

**FIGURE 16:**
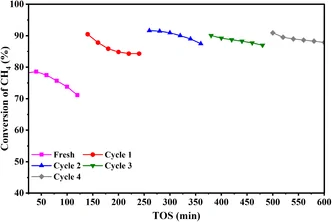
CH_4_ conversion of regenerated Fe‐10ZrAl catalyst across 4 cycles at 800 °C, *p* = 1 atm, GHSV = 3600 mL h^−1^g_cat_^−1^, and CH_4_:N_2_ ratio of 2:1.

To investigate the regeneration ability and long‐term stability of the Fe‐10ZrAl catalyst, multiple cycles of methane decomposition‐regeneration were conducted. A freshly prepared Fe‐10ZrAl catalyst was used for this process. In the oxidative regeneration, the accumulated carbon was removed by treatment with O_2_ at 850 °C for 50 min. The evolution of CO and CO_2_ during the oxidative regeneration process was continuously monitored until the signals returned to the baseline level. In the following methane decomposition step, there should be no CO or CO_2_ (within the detection limits of GC) after N_2_ purging**.** As illustrated in Figure 16, the initial CH_4_ conversion rate increased markedly from 78.1% for the fresh catalyst to 90.9% within the fourth cycle, demonstrating that the regeneration process not only restored but also enhanced catalytic activity, likely due to the structural reorganization of the Fe active sites and an increase in metal dispersion occurring during the repeated decomposition‐regeneration steps. Even though the conversion showed a noted gradual decline in each reaction cycle, the degree of deactivation diminished progressively with each regeneration. Notably, the conversion loss per cycle decreased from 6.28% in the first cycle to only 3.3% by the fourth cycle, indicating a significant enhancement in catalyst stability post‐regeneration. This further confirms the thermal stabilizing role of ZrO_2_ observed initially, as is common in the literature [32]. Raman spectroscopy was used to examine the structural properties of the deposited carbon on the Fe‐10ZrAl catalyst after the fourth regeneration cycle. A reference Fe‐10ZrAl sample from the reaction of the previous catalyst batch under identical conditions was used for comparison, as shown in Figure S5. The regenerated catalyst displayed a notably lower *I*_D_/*I*_G_ ratio of 0.64 compared to the reference of 0.87, suggesting that the regeneration process effectively facilitated the formation of more ordered graphitic carbon (Figure S5A). The spent Fe‐10ZrAl sample after four cycles showed greater mass loss (65.1%) than the spent Fe‐10ZrAl catalyst (mass loss 56.2%) (Figure S5B). Interestingly, the mass loss in the Fe‐10ZrAl sample after four cycles initiates at a higher temperature. It indicates the presence of such carbon, which is more inert/graphitic carbon, over the catalyst surface. XRD analysis of spent Fe‐10ZrAl catalyst shows separate phase for graphitic carbon (at 26.2° Bragg's angle; JCPDS reference number 96‐101‐1061) and metallic cubic Iron phases (at 43.7°, 44.6°, 50.9°, 74.7° Bragg's angle; JCPDS reference number 96‐900‐8470 and 96‐900‐8537) (Figure S5C). It can be concluded that the metallic iron phase and graphitic carbon phase are distinctively separated from each other in successive regeneration cycles. In that sense, the masking possibility of iron by carbon is neglected over the Fe‐10ZrAl catalyst after successive regeneration. During regeneration under oxygen, some Fe species may be oxidized to iron oxides. In the subsequent reaction cycle, the catalyst is likely reactivated under methane decomposition conditions via in situ reduction of iron oxides to active metallic Fe. The sequential oxidation of carbon deposited under oxygen and in situ reduction under reaction conditions maintain high catalytic performance across cycles. Thus, the Fe‐10ZrAl catalyst demonstrates high activity retention and powerful regeneration capability, positioning it as a promising candidate for cyclic methane decomposition to produce hydrogen and carbon nanomaterials. The regeneration route can be further understood from a textural and phase‐evolution perspective to understand the Fe‐10ZrAl catalyst outstanding structural robustness across many operating cycles. During regeneration, the interconnected carbon tubular network is subjected to an oxidative treatment step, during which oxidative gasification is performed to clean the active metal surface. Simultaneously, there is a temporary oxidation of the active iron phase. This process must be controlled by the presence of the modifying oxide support (e.g., ZrO_2_) via strong metal–support interactions that anchor and stabilize the iron nanoparticles against thermal sintering and the formation of chemically inert deep‐lattice bulk oxides. The methane feed initiates an in‐situ reduction that quickly transforms the surface iron oxides into active metallic iron Fe^0^ and re‐generates the necessary Fe_3_C (cementite/cohenite) intermediates required for the carbide cycle mechanism. Post‐regeneration XRD analysis clearly demonstrates the structural integrity, with a perfect recovery of well‐defined cubic iron phases without any structural degradation. However, a thorough post‐reaction XRD and TEM analysis was performed on the spent samples to track the structural changes. The studies confirmed that active metallic iron (Fe^0^) is converted to an iron carbide (Fe_3_C) intermediate phase upon contact with methane. Moreover, the catalytic stability profile shown in Figure 16 shows a significant deviation from the rapid deactivation usually observed in conventional methane decomposition systems, where continuous amorphous carbon deposition rapidly covers active metal sites. In our catalyst system, this deactivation is relieved by a continuous “carbide cycle” process. Methane dissociates at the active sites, and the resulting carbon atoms diffuse rapidly into the iron matrix, forming a transient phase of Fe_3_C. Once supersaturation is obtained, the carbon is continuously expelled outward as crystalline, graphitic MWCNTs. This carbon growth behavior, consistent with the previously observed base‐growth mode of Fe‐10ZrAl, contributes to maintaining the accessibility of active Fe sites during repeated cycles.

## 6 Conclusions

This study involved the synthesis of iron‐supported catalysts via wet impregnation, which were subsequently assessed in CDM. Fe‐based catalysts that were directly supported on Al_2_O_3_, ZrO_2_, and SiO_2,_ as well as those supported on modified Al_2_O_3_ by 10% of SiO_2_, ZrO_2_, CeO_2_, or Y_2_O_3,_ were assessed. Every catalyst was tested for 120 min on stream at 800 °C. The comparative analysis and results over all catalysts are presented in Figure S6. A catalyst must possess a sufficient surface area to carry active sites for a reaction. Among 20Fe/Al_2_O_3_ (Fe‐Al), 20Fe/SiO_2_ and 20Fe/ZrO_2_, only Fe‐Al possesses a good surface area as well as good CH_4_ conversion (69‐20%) during 120 min on stream. The fast drop of activity over Fe‐Al is due to the severe iron sintering and graphitic carbon deposition. The next catalytic development is achieved by modifying the support (alumina) with 10 wt.% of a metal oxide (M = Zr, Y, Ce, Si), which retains a high surface area (≥150 m^2^/g), larger Fe crystallite sizes than the Fe‐Al catalyst with the exception of that of Fe‐10YAl, and demonstrated excellent reducibility. Among metal–oxide modified catalysts, ZrO_2_ addition brings the highest abundance of reducible species, confirming prominent C·H dissociation and catalyst excellence. The successive regeneration of the Fe‐10ZrAl catalyst also leads to distinct phase separation of metallic Fe and graphitic carbon, thereby reducing the chance of carbon encapsulation of the active site and retarding activity. Overall, the fresh Fe‐10ZrAl catalyst achieves 76.5%–68.5% CH_4_ conversion and 74.1%–66.2% H_2_ yield during 120 min. Most importantly, the ZrO_2_‐modified catalyst outperforms after the fourth regeneration, achieving about 90% CH_4_ conversion. The spent Fe‐10ZrAl catalyst also exhibited the most uniform distribution of CNT diameters. The Y_2_O_3_‐modified catalyst and SiO_2_‐modified catalyst have adequate surface area as well as a comparable abundance of reducible species, but the former attains the highest active phase crystallite size. So, the activity of Fe‐10YAl is better (than Fe‐10SiAl) and it retains ∼59% CH_4_ conversion and 56% H_2_ yield at the end of 120 min time on stream. Over Ce‐modified catalysts, hydrogen consumption may, in part, be due to ceria reduction. Overall, the catalytic activity performance of Fe‐10CeAl and Fe‐10SiAl towards methane decomposition is poor, and they retain less than 25% H_2_ yield at the end of 120 min of reaction.

## Author Contributions

**Hajer S. Alturki, Mohammed O. Bayazed, and Ahmed A. Ibrahim**: data curation, writing, formal analysis, methodology, writing – review and editing. **Abdulaziz A. M. Abahussain and Naif Alarifi**: investigation, formal analysis, methodology. **Fahad S. Almubaddel, Seham S. Alterary, and Alaaddin M. M. Saeed**: characterization, resources, visual. **Rawesh Kumar**: writing – review and editing. **Ahmed I. Osman and Ahmed S. Al‐Fatesh**: conceptualization, writing, reviewing and editing, funding acquisition, project Administration.

## Funding

This study was supported by King Saud University (Ongoing Research Funding program (ORF‐2026‐368), King Saud U).

## Conflicts of Interest

The authors declare no conflicts of interest.

## Supporting information

The authors have cited additional references within the Supporting Information [61, 62, 63, 64, 65, 66, 67, 68, 69].

## Data Availability

All data that support the findings of this study are included within the article.
