# Supplementary material for: Iron‐Based Catalysts on Tailored Alumina Supports for CO x ‐Free Methane Decomposition to H2 and Carbon
Source: ChemistryOpen. 2026 Sep 1;15(9):e70282. doi: 10.1002/open.70282 (PMC13533779; doi:10.1002/open.70282)
Supplement: Supplementary file 1 [file OPEN-15-e70282-s001.pdf]

Iron-Based Catalysts on Tailored Alumina Supports for CO_x-Free MethaneDecomposition to H_2

Hajer S. Alturki[1], Seham S. Alterary[1], Abdulaziz A. M. Abahussain[2],Ahmed I. Osman[3*], Mohammed O. Bayazed[2], Ahmed A. Ibrahim[3], Naif Alarifi[4], Fahad S. Almubaddel[2],Alaaddin M. M. Saeed[5], Rawesh Kumar[6], Ahmed S. Al-Fatesh[2]*

[1]Department of Chemistry, College of Science, King Saud University, P.O. Box. 2455, Riyadh, 1145, Saudi Arabia
[2]Department of Chemical Engineering, College of Engineering, King Saud University (KSU), P.O. Box 800, Riyadh 11421, Saudi Arabia
[3]School of Sciences, Psychology, Arts and Humanities, Computing, Engineering & Sports, Canterbury Christ Church University, Canterbury CT1 1QU
[4]Institute of Refining and Petrochemicals Technologies, King Abdul Aziz City for Science and Technology (KACST), Chemistry, Faculty of, Riyadh 11422, Kingdom of Saudi Arabia
[5]UNILAB, State Key Laboratory of Chemical Engineering, East China University of Science and Technology, Shanghai 200237, China.
[6]Department of Chemistry, Patna University, Bihar, India, 800005
* Correspondence: ahmed.osman@canterbury.ac.uk (A.I.O); aalfatesh@ksu.edu.sa (A.S.A.-F.)

Catalyst and carbon characterization S1

The catalysts' textural characteristics were assessed using a Micro-meristic Tristar II 3020 surface area and porosity analyzer at -196°C. Before analysis, 0.2 g of each sample was degassed for three hours at 250 °C to eliminate any remaining moisture content and other adsorbed gases from the catalyst surface. The Brunauer-Emmett-Teller (BET) and Barret-Joyner-Halenda (BJH) techniques were used to determine the specific surface area and pore volume of the catalyst samples, respectively.

The AutoChem II 2920 chemisorption analyzer (Micromeritics, USA) was utilized for temperature programmed reduction (TPR). 70 mg of the catalyst samples were placed in

a tube integrated into the machine's furnace to initiate the TPR process. The samples were pretreated by flushing them with argon at 150 °C for 60 min, after which they were permitted to reach room temperature. Then, an H_2/Ar mixture flowed at 40 mL/min while the furnace temperature increased to 1000 °C at 10 °C/min. The machine's internal cold trap removed the water generated during the reduction, and a thermal-conductivity-based detector tracked the amount of H_2 consumed.

X-ray diffraction (XRD) analysis was utilized to investigate the crystal structure and phase formation of the catalyst samples. The examination was conducted using a Miniflex Rigaku diffractometer equipped with Cu Ka X-ray radiation, operating at 40 kV and 40 mA. The diffraction 2θ angle range was set to 10-80. The raw data files from the instrument were analyzed using X'PertHighScore Plus software. The identified phases and their corresponding scores were compared against the JCPDS database for matching. The Scherrer equation has been used to calculate the crystal size ($D = \frac{k\lambda}{(\beta \cos\theta)}$; where k, λ, β, and θ are the Scherrer constant, wavelength, Full width at half maximum (FWHM) of the peak, and Bragg angle (θ).

A Transmission Electron Microscope (TEM), JEM-2100F, operating at 200 kV, was used to conduct a thorough examination of pristine and nanostructured carbon deposits on spent catalysts. TEM images were captured.

Thermogravimetric analysis (TGA) was employed to quantitatively analyze the formed carbon on the used catalysts. Weight loss was evaluated when 10-15 mg of the utilized catalyst was heated from 50 to 1000 °C at a ramp rate of 20 °C/min in an air atmosphere. With the aid of a JASCO laser Raman spectrometer (NRS-4500) from Tokyo, Japan, the

Catalyst name	Conversion %		Yield %		S_{BET} (m^2/g)[a]
	$CH_{4\ initial}$	$CH_{4\ final}$	$H_{2\ initial}$	$H_{2\ final}$	
20Fe/Al_2O_3	69.4	19.5	67.1	18	160

degree of graphitization and the kind of carbon deposited over the utilized catalysts were ascertained. The beam excitation wavelength used by the spectrometer is 532 nm. A 100× magnification and a 1.6 mW laser intensity were used in this measurement. To construct each spectrum, three exposures within ten seconds were averaged. Eventually, the spectra, recorded within a shift range of 1200–3000 cm^{-1}, were processed via Spectra Manager Ver. 2 software.

X-ray photoelectron spectroscopy (XPS, Thermo Fisher Scientific, USA) with Al Kα radiation was employed to investigate the oxidation states of elements and surface enrichment of metal oxides. For charge correction, a one-point scale with C (1s) peak shifted to 285.0 eV was used

Table S1: Effect of Catalyst Support on Methane Conversion and Hydrogen Yield

20Fe/ZrO_2	3.6	2.7	3.0	2.5	9
20Fe/SiO_2	4.7	4.6	4.1	3.9	6

a: Surface area for fresh catalysts

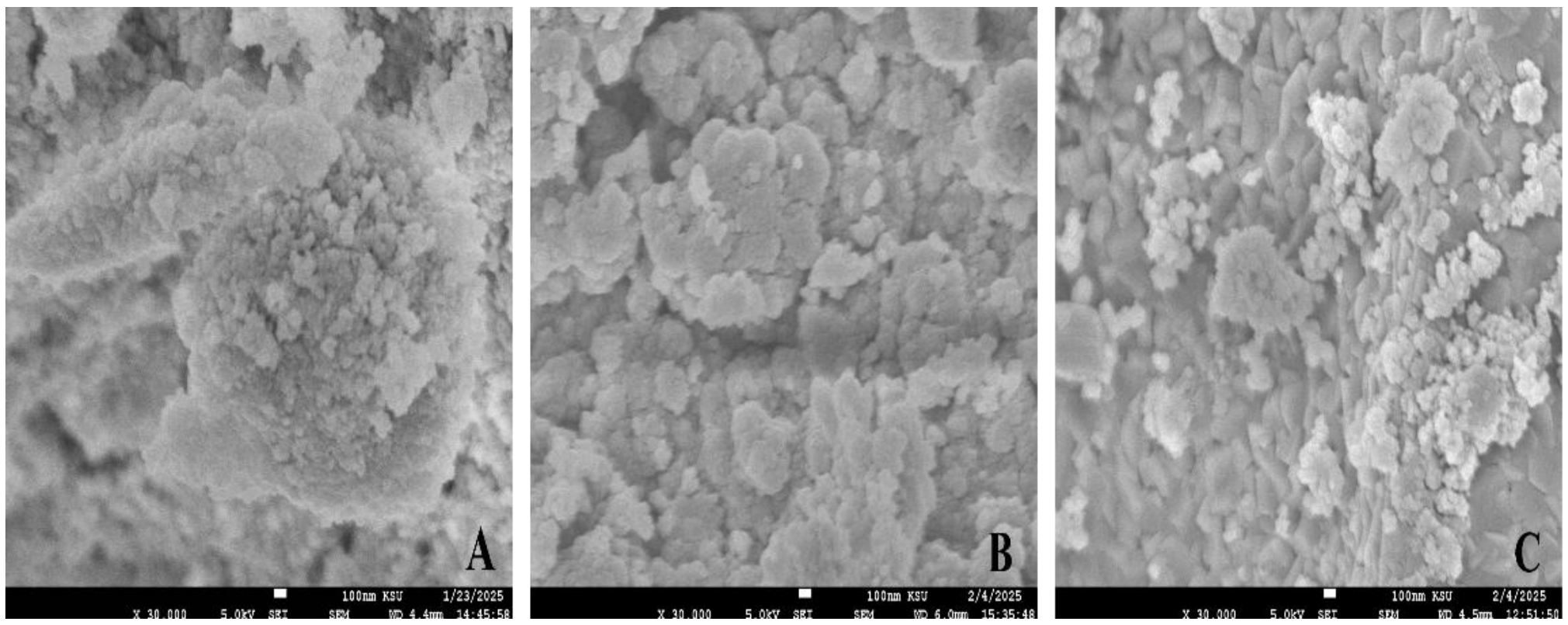

Figure S1 SEM of fresh samples (A) Fe/Al_2O_3, (B) Fe/SiO_2, (C) Fe/ZrO_2

Table S2: Textural properties for catalystsbefore and after reaction

Catalyst name	Catalyst Abbreviation	SA (m^2/g)		PV (cm^3/g)		PS (nm)	
		Fresh	Spent	Fresh	Spent	Fresh	Spent
20Fe/Al_2O_3	Fe-Al	160	119	0.51	0.42	11.3	14.6
20Fe/(10%CeO_2+90%Al_2O_3)	Fe-10CeAl	157	137	0.50	0.46	11.2	14.1
20Fe/(10%SiO_2+90%Al_2O_3)	Fe-10SiAl	150	119	0.46	0.48	11.2	17.0
20Fe/(10%ZrO_2+90%Al_2O_3)	Fe-10ZrAl	150	113	0.45	0.48	10.9	17.4
20Fe/(10%Y_2O_3+90%Al_2O_3)	Fe-10YAl	164	125	0.48	0.46	10.4	14.9

Note: SA: Surface area, PV: Pore volume, PS: Average Pore size

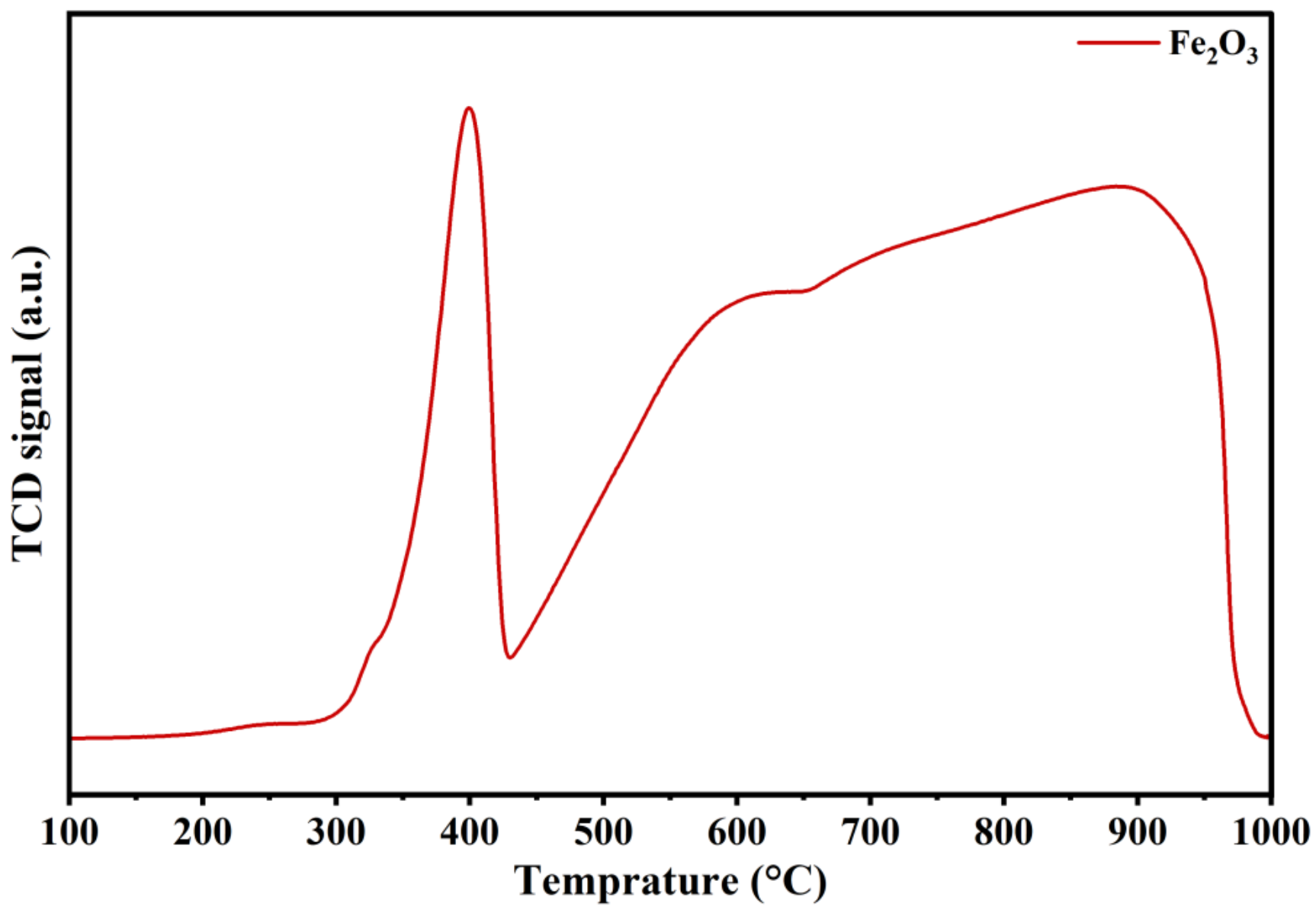

Figure S2. H_2-TPR patterns for fresh pure Fe_2O_3

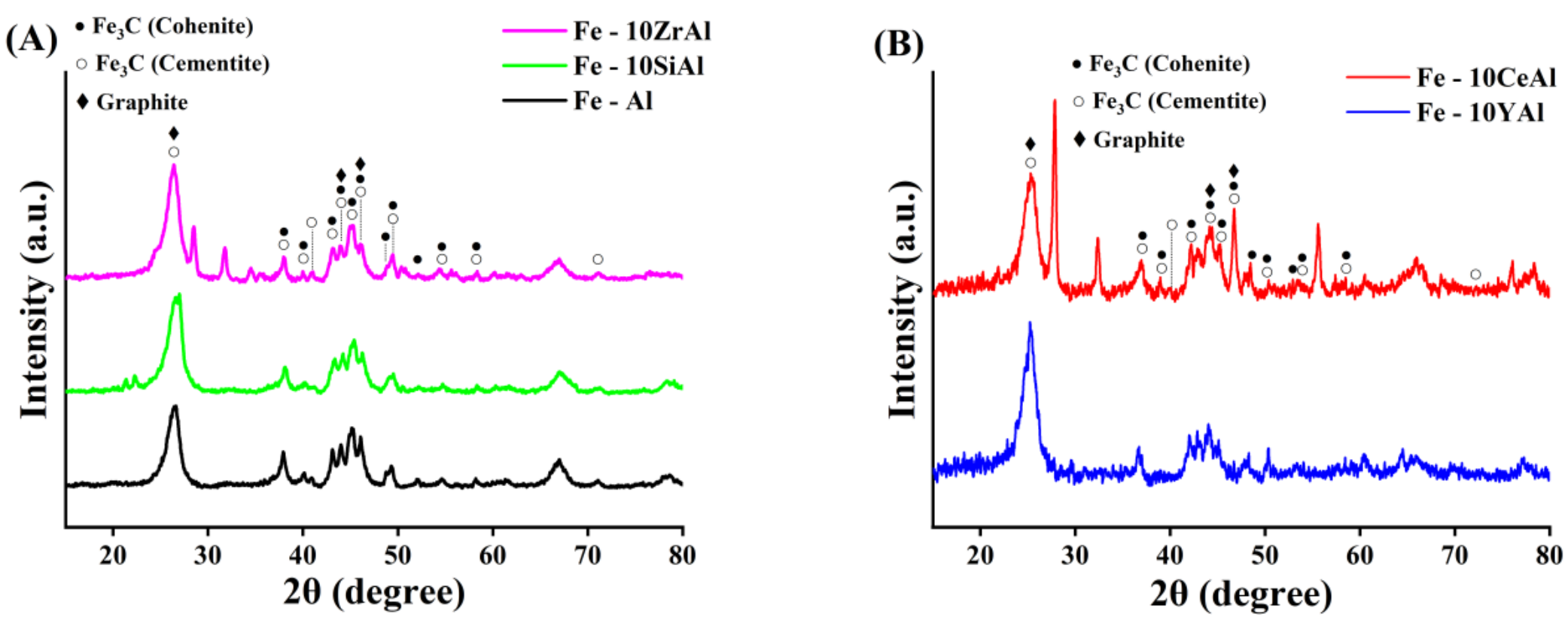

Figure S3. XRD patterns of spent Fe-based catalysts after 120 min of reaction. (A) Fe-10ZrAl, Fe-10SiAl, and Fe-Al catalysts. (B) Fe-10CeAl and Fe-10YAl catalysts.

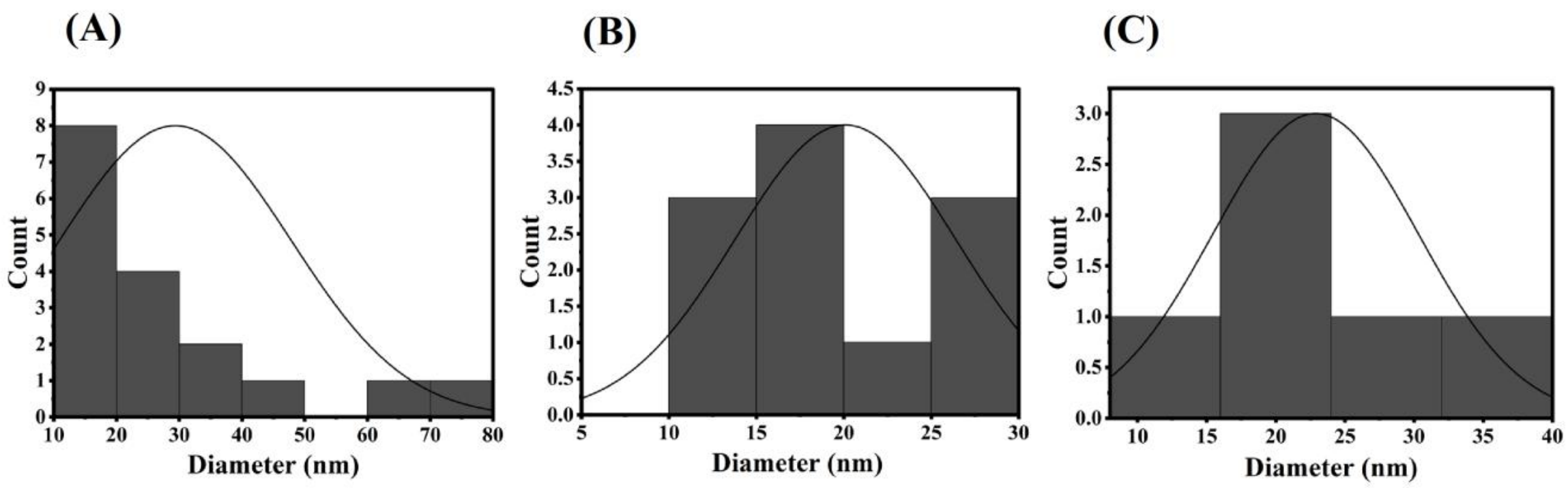

Figure S4. Diameter distribution of CNTs (from TEM images): (A) Fe-Al, (B) Fe-ZrAl, and (C) Fe-YAl.

Table S3: Physicochemical Properties and Relative abundance of Reducible species of Fe-Based catalysts

**Catalyst**	**Crystallite Size (nm)**	Relative abundance of Reducible species	**S_{BET} (m^2/g)**
Fe-Al	12.7	Moderate	160
Fe-10SiAl	14.9	Lowest	150
Fe-10CeAl	21.7	Low	157
Fe-10YAl	7.2	High	164
Fe-10ZrAl	13.8	Maximum	150

Relativeabundance of reducible speciesis based on H_2-TPR intermediate reduction peak areas

Table S4: Comparison of this work activity with previous literature results.

Catalyst	Rx. temp. (°C)	TOS (min)	Conversion (%)		Yield %		Ref
			$CH_{4\ initial}$	$CH_{4\ final}$	$H_{2\ initial}$	$H_{2\ final}$	
$20Fe/Al_2O_3$	700	180	47	65	45	60	[18]
$60\%Fe/Al_2O_3$	700	60	58	20	26	12	[65]
50%Fe/MgO	700	570	-	-	12	45	[66]
$20\%Fe/\ ZrO_2$	800	240	60	20	-	-	[22]
$20\%Fe/La_2O_3+ZrO_2$	800	240	80	25	-	-	
$15\%Fe/CeZrO_2$ ($CeO_2:ZrO_2$ = 1:5.25)	700	125	85	36	83	33	[61]
$20\%Fe/TiO_2$-Al_2O_3 ratio (wt%: 20:80)	800	240	-	-	52	81	[28]
$20\%Fe/CeO_2$-Al_2O_3 ratio (wt%: 20:80)	800	240	85	53	79	37	[67]
Fe_2O_3/Y_2O_3(1:1)	800	236	14	6	14	6	[68]
$Fe_2O_3/Y_2O_3/Al_2O_3$(3:1:6)	800	500	4.2	3	4.2	3	
$75Fe/SiO_2$	800	240	34	1	-	-	[20]
$20\%Fe/10SiO_2+90Al_2O_3$	800	300	74	48	-	-	[69]
$30\%Fe/Y_2O_3+ZrO_2$	800	180	69	50.1	-	-	[64]
Fe-10YAl	800	120	73.7	58.6	71.3	56.3	This work
Fe-10ZrAl	800	120	76.5	68.5	74.1	66.2	This work

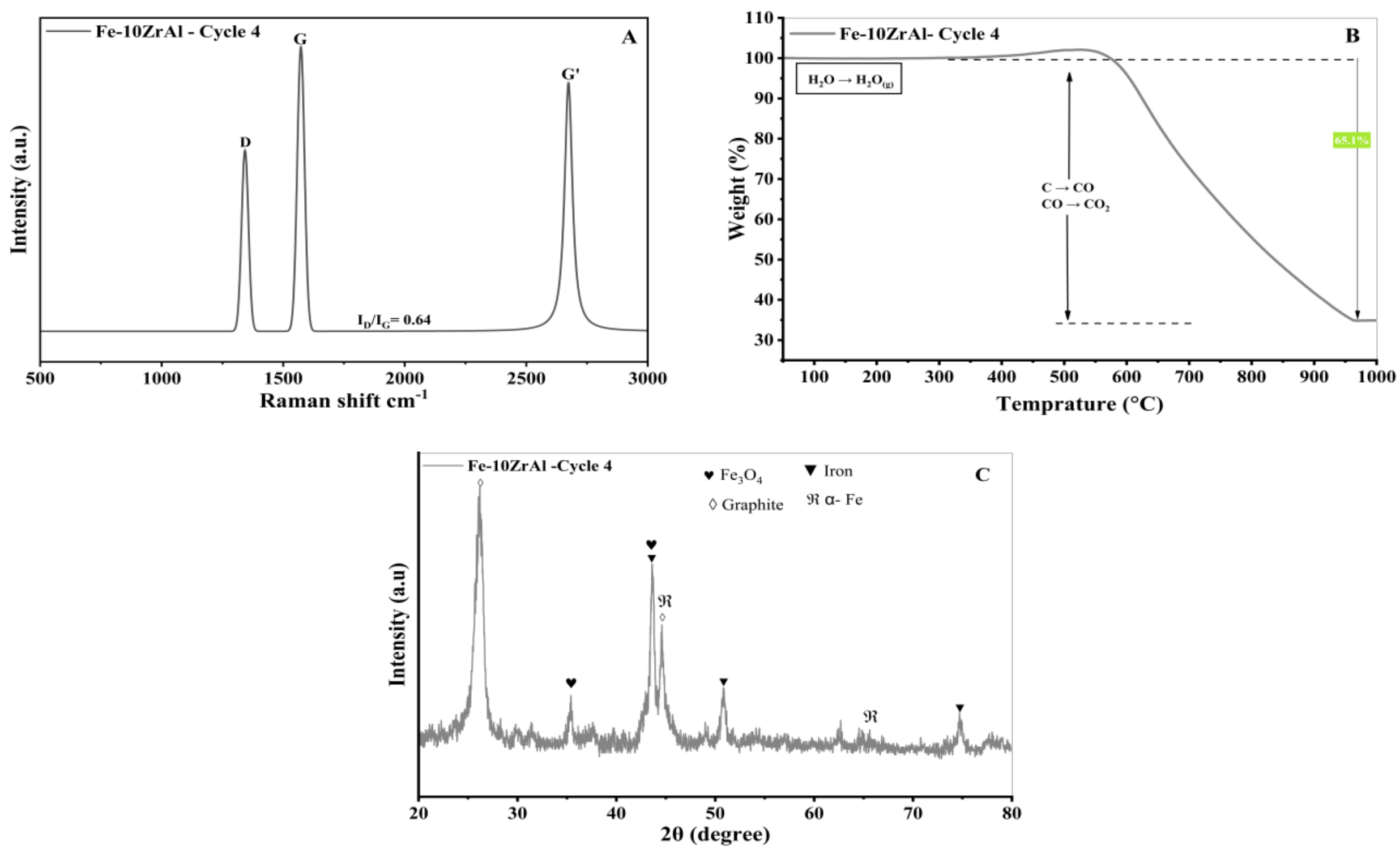

Figure S5. Characterization of spentFe-10ZrAl catalyst after the fourth regeneration cycle, 600 min of reaction at 800 °C(A) Raman spectra (B) Thermogravimetry, , (C) X-ray diffraction.

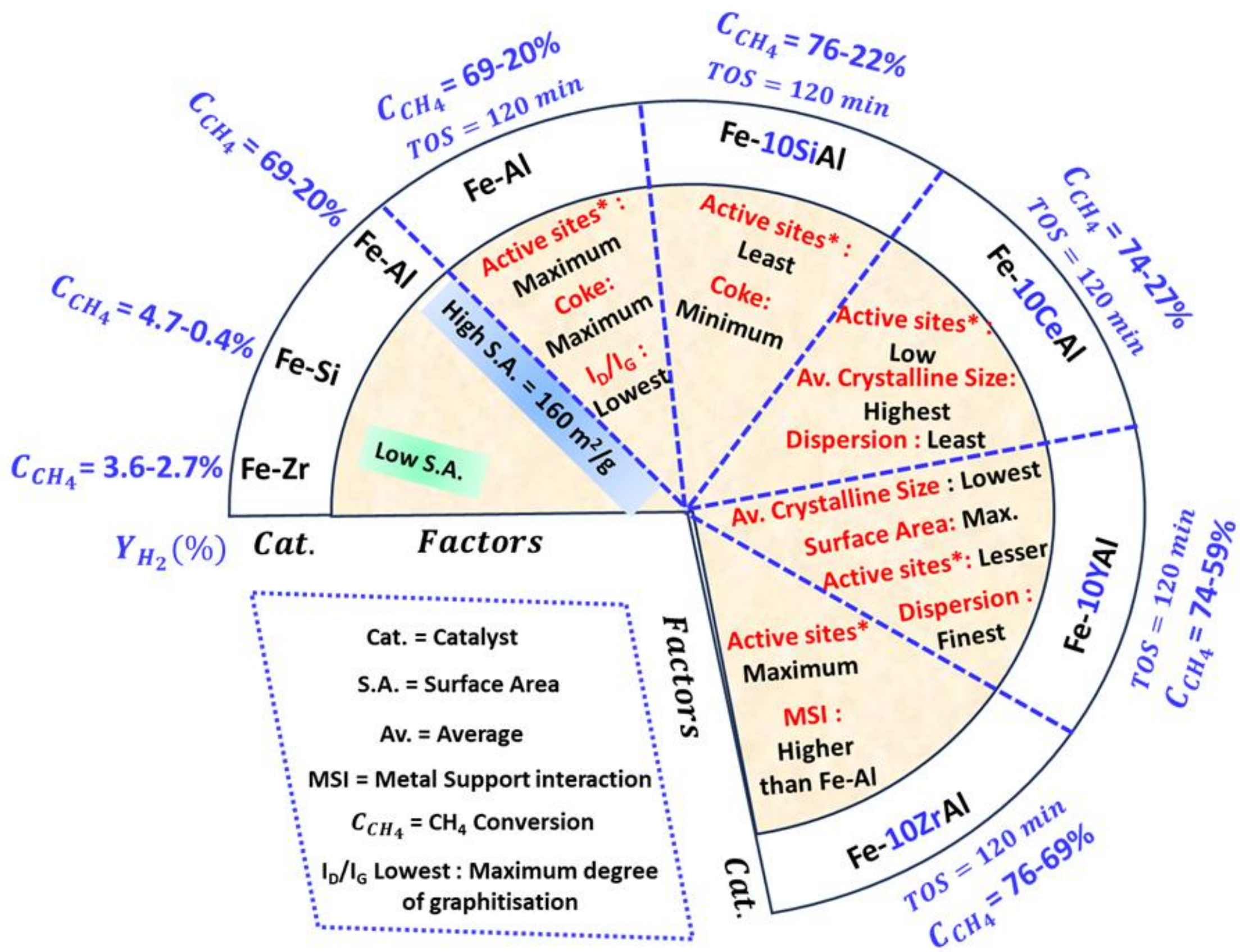

Figure S6. The primary findings of the catalytic activity and characterization data for the various catalysts under investigation. Reducible species abundance is based on the reduction peakareas in the intermediate temperature range of H_2-TPR.
